# Photocatalytic degradation of perfluorooctanoic acid under ambient conditions validated by duckweed as a sensitive ecotoxicity assay

**DOI:** 10.1016/j.hazmo.2026.100016

**Published:** 2026-02-12

**Authors:** Wan Zhang, Meichen Wang, Vincent Xu, Johnson O. Oladele, Yohannes H. Rezenom, Timothy D. Phillips, Susie Y. Dai

**Affiliations:** a Department of Plant Pathology and Microbiology, Texas A&M University, College Station, TX 77843, United States; b Department of Environmental Health Sciences, University of Massachusetts Amherst, Amherst, MA 01003, USA; c Department of Energy, Chemical and Environmental Engineering, Washington University in St Louis, MO 63130, United States; d Department of Veterinary Physiology and Pharmacology, College of Veterinary Medicine & Biomedical Sciences, Texas A&M University, College Station, TX 77845, USA; e Department of Chemistry, Texas A&M University, College Station, TX 77843, United States; f Department of Chemical and Biomedical Engineering, University of Missouri, Columbia, MO 65211, United States

**Keywords:** Photocatalysis, Toxicological Assessments, PFAS, Duckweed

## Abstract

Photocatalytic degradation of perfluorooctanoic acid (PFOA) has drawn great attention in the past. These studies have focused on developing high-efficacy photocatalysts and understanding the reaction mechanisms and structure-performance relationships. However, the photocatalytic degradation efficacy and kinetics under environmentally relevant conditions and the reduced toxicological impact of degraded PFAS products are lacking. To fill in this gap, we developed a photocatalytic system to deconstruct perfluorooctanoic acid (PFOA) under ambient conditions using sunlight as a renewable energy source. We evaluated the degradation pathway and reaction mechanisms using high-resolution mass spectrometry (LC/MS-MS) and product portfolio. The photocatalytic degradation of PFOA in environmental water matrices was set up over a two-week period using natural sunlight outdoors. The toxicity of degraded products was assessed in parallel using duckweed, or *Lemna minor* as a representative ecotoxicological assay during the same time course. Degraded products containing shorter carbon chain PFAS such as perfluoroheptanoic acid (PFHpA, C7) and perfluorohexanoic acid (PFHxA, C6) showed significantly reduced toxicity to *L. minor*, compared to untreated PFOA water. In the day and night cycles of PFOA photocatalytic treatment using natural sunlight, the measured toxicity of the photocatalytic reaction system continued to decline during the two-week period. However, the concentrations of measured PFAS, including degraded products of PFHpA and PFHxA, remained stable after six days for a period of 2 weeks. Our study suggested another potential detoxification mechanism exists with prolonged treatment, which leads to continuously reduced toxicity. Our results highlighted the need for a systematic approach combining analytical chemistry for degradation mechanisms and ecotoxicological models to perform time-dependent toxicological assessments of PFAS destruction.

## 1. Introduction

The presence of poly- and perfluoroalkyl substances (PFASs) in our environment, such as drinking water sources and wastewater, is of increasing concern due to their extreme persistence and toxicity [1–3]. However, PFAS have been produced in large quantities since the 1950s, which presents a significant environmental challenge. For example, Perfluorooctanoic acid (PFOA) has been used as a surfactant in the manufacture of fluoropolymers. Between 1951 and 2004, the estimated total global production of PFOA and its ammonium salt (APFO) ranged from 3600 to 5700 tons [4]. Various technologies have been developed to remove and deconstruct important PFAS, such as PFOA, to reduce human exposure to the toxic compound and protect environmental health [5]. Among various PFAS deconstruction methods, photocatalytic degradation of PFAS is a popular approach because photocatalysis can be operated under mild operation conditions and is environmentally friendly [6]. The defluorination and degradation pathways of PFAS largely depend on the dominant reactive oxidizing or reducing species involved in the system. For instance, photoinduced holes typically oxidize PFAS into unstable perfluoroalkyl radicals by removing head groups, whereas hydroxyl and superoxide radicals are capable of cleaving C-C and C-F bonds. In contrast, hydrated electrons can directly eliminate the head group, followed by stepwise release of CF_2_ units [7]. As a result, long-chain PFAS such as PFOA are gradually degraded into shorter-chain intermediates, for example, PFOA → PFHpA → PFHxA. While the majority of the photocatalytic degradation of PFAS has been focused on using UV light, we have recently developed a lignin-doped 3D shaped photocatalyst that can efficiently deconstruct PFOA using sunlight at ambient conditions [8]. Using sunlight in the visible light wavelength range, the lignin-doped TiO_2_ photocatalyst can degrade PFOA into PFHpA and PFHxA within 30 min, providing an ideal model scenario to simulate sunlight to deconstruct PFOA in the natural environment.

It has been commonly accepted that PFAS deconstruction will effectively reduce toxicity and short-chain PFAS molecules are typically less toxic than long-chain PFAS [9,10]. According to the definition proposed by Buck et al. [11], long-chain perfluorocarboxylic acids (PFCAs) include those with eight or more perfluorinated carbons (≥ C8), such as PFOA, whereas short-chain PFCAs contain seven or fewer carbons (≤ C7). Thus, most of the PFAS deconstruction studies focus on converting PFAS into shorter-chain degradation products and ultimately reaching mineralization. Indeed, there is a large scientific literature reporting the PFAS libraries and summarizing individual PFAS toxicity data [12,13]. It is more complicated to evaluate PFAS mixtures even though the cumulative toxicity of PFAS mixtures is more relevant to real-life conditions. In a PFAS degradation system, the degraded products represent dynamic PFAS mixtures, whose profiles change over time. As such, continuous ecotoxicological evaluation of degraded products generated in a PFAS photocatalytic degradation system can be an essential tool for assessing treatment outcomes. This approach provides more environmentally relevant insights into the ecological and health-related impacts of PFAS in natural ecosystems. However, to our best knowledge, no prior study has evaluated the dynamic toxicological impacts of a reactive degradation system. Particularly, the scientific basis for managing PFAS by degradation is that short-chain PFAS is less toxic than long-chain PFAS. Therefore, it is necessary to understand the detoxification process and evaluate the toxicity of PFAS-degraded products over time.

Many ecotoxicological models have been used to evaluate PFAS toxicity in water, such as fish, amphibia, algae, Mollusca, crustacea, Chironomus, and plants [14]. Among the plants, *Lemna minor* (or duckweed) is a well-established model organism for ecotoxicity assessment and is significantly relevant to the study of freshwater systems. *L. minor* can be used to detect a wide range of toxins at low concentrations, with multiple toxicological endpoints based on growth, function, and morphology including frond number, growth rate, and chlorophyll content. It represents a sensitive assay to evaluate the effectiveness of PFAS degradation in terms of toxicity over time, with low cost and minimum maintenance requirements. Previous studies have used *L. minor* to evaluate PFAS toxicity such as Perfluorooctanesulfonamide (PFOSA), 6:2 fluorotelomer sulfonate (FTSA) and PFOA. Thus, we selected *L. minor* to evaluate the toxicity of complex PFAS mixtures before and after PFOA degradation. [15,16]. Given the capacity of *L. minor* to deliver various responses with different PFAS at the same concentration, *L. minor* was selected to evaluate the toxicity of complex PFAS mixtures before and after PFOA degradation.

Based on our recent findings, the lignin-based photocatalyst can serve as an ideal model to evaluate a reactive PFAS degradation system under ambient conditions [8]. Using natural sunlight as an energy source, we evaluated the PFOA degradation products and toxicological impacts on *L. minor* over time. Based on the literature review [17–19], *L. minor* is used as an indicator for PFAS toxicity in this study due to its ability to predict the toxicity of chemical contaminants in water. *Lemna* is an aquatic, free-floating plant that can absorb toxic chemicals from water. Since PFAS is amphipathic, soluble in water and persistent in the environment, Lemna readily interacts with PFAS and exhibits visible changes in growth, frond number reduction and chlorosis that are associated with toxicity. Importantly, *L. minor* is well-recognized in OECD and EPA guidelines for use in aquatic toxicity testing, ensuring reproducibility of the data and regulatory acceptance. The objectives of this study were twofold: 1) to investigate the PFOA photocatalytic degradation reaction and delineate degradation mechanisms, and 2) to evaluate the safety and efficacy of photocatalysis using a PFAS-sensitive ecotoxicological model. Our work assessed and validated PFOA degradation and detoxification in various environmental water matrices using kinetic models, and these suggested that photocatalytic degradation of PFAS could be a viable way to remediate PFAS in the natural environment.

## 2. Methods

### 2.1. Photocatalyst synthesis

The photocatalyst was synthesized [8] according to our previous study. Colloidal lignin particles were first initiated by dissolving 2 g of kraft lignin (dry basis) in 200 mL of an acetone/water 3:1 (v/v) mixture and stirred for 3 h, followed by filtration using a glass microfiber filter (Titan3^™^ Glass Microfiber Syringe Filters, Thermo Fisher Scientific, pore size 0.7 μm) to remove the undissolved lignin. The obtained solution was rapidly poured into 400 mL of Milli-Q water under vigorous stirring for 3 h. Acetone was further removed by reduced pressure distillation at 40 °C to obtain the lignin nanoparticle dispersion. The dispersed lignin nanoparticles were freeze-vacuum dried and then stored in a desiccator for further characterization and use. To prepare lignin@H-TiO_2_, 340 mg titanium (IV) *n*-butoxide and 4 mg lignin nanoparticle was added to 50 mL ethanol with magnetic stirring for 3 h until a clear brown uniform solution was obtained. Subsequently, 10 mL Milli-Q water was added dropwise to the above solution, which gradually turned into an opaque brown-yellow dispersion due to the hydrolysis reaction of titanium (IV) *n*-butoxide. The opaque brown-yellow solution was left to react for 12 h, ensuring complete hydrolysis of the titanium (IV) *n*-butoxide and its successful copolymerization with lignin. After the reaction, the dispersion was centrifuged with ethanol twice and Milli-Q water five times to remove any unreacted lignin and residual ethanol. The resulting precipitate was subsequently dried at 60 °C for 3 h, yielding brown powder identified as lignin@H-TiO_2_. The lignin@H-TiO_2_ samples were calcined into C_lignin_@H-TiO_2_ in a split tube furnace with a vacuum system (GSL 1600X, MTI Corporation, Richmond, CA). The thermostabilization process was carried out with heating from room temperature to 200 °C at a heating rate of 5 °C min^−1^ and then holding at 200 °C for 20 min. Afterwards, the thermostabilized lignin@H-TiO_2_ was carbonized under a nitrogen atmosphere (240 cm^3^ min^−1^). The temperature was increased from 200 °C to 600 °C with a heating rate of 5 °C min^−1^. The holding time at calcination temperature was 1.5 h. After the calcination process, a black powder was obtained as C_lignin_@H-TiO_2_.

### 2.2. Photocatalytic treatment

The performance of photocatalytic degradation was first studied at laboratory scale, 10 mg C_lignin_@H-TiO_2_ was added in 5 mL porcelain crucibles with 5 mL PFAS solution (HPLC grade water). After 5 min adsorption equilibrium, the porcelain crucibles were placed under a solar simulator equipped with a xenon lamp (CME-SL500, Microenerg Beijing Technology Co., Ltd) with an intensity of 1800 m^−2^ and a working distance of 15 mm to test the photodegradation performance. After a certain time of light treatment, sample solutions were collected from the porcelain crucibles and filtered through a 0.2 μm filter into volumetric flask, then HPLC grade water was added into the above collected sample solutions to 5 mL. For the *L. minor* assay toxicity evaluation, the *L. minor* growth media was added into the above collected sample solutions to 5 mL.

The performance of photocatalytic degradation under sunlight was also studied from September 25 to October 6, 2024 at the Texas A&M campus. 500 mg C_lignin_@H-TiO_2_ was added in 250 mL PFAS solutions in plastic bottles. After 30 min adsorption equilibrium, the plastic bottles were placed under sunlight for 16 consecutive days. 10 mL solution was collected at day 0, 3, 6, 9, 12, 15 and 16, and filtered for mass determination and toxicity evaluation in *L*. *minor* assay. HPLC grade water (Thermofisher), laboratory tap water, groundwater collected in Texas (Starr County, Private well, TX), rainwater collected in College Station (Latitude 30.548292, Longitude −96.277508) and wastewater collected at Texas A&M University wastewater treatment plant were used separately to prepare the 250 mL PFAS containing solutions.

### 2.3. L. minor assay and toxicity evaluation

The *Lemna minor* was purchased from AquaHabit (Chatham, England) adapted and maintained in a mineral media based on previous reports [20,21]. The standard EPA guideline OCSPP 859.4400 [22] was followed for the toxicity assay with the following modifications: a lower radiation with cool white, fluorescent lights was used as the light source (400 ft-c intensity) and the mineral media was used to sustain the *L. minor* culture during the toxicity test [20,21]. The plant was cultured with cool white fluorescent lights (400 ft-c intensity) at a light-to-dark cycle of 16 h/8 h and a mean temperature of 25 °C in a mineral growth medium [21]. Three colonies of 3-frond *Lemna* plants were randomly selected and incubated in Pyrex dishes closed with loose-fitting lids for 7 days. *Lemna* was exposed to varying doses of PFOA, PFHxA, and PFHpA at 1, 5, 7.5, 10, 20, 30, 40, 50, 110, 150, 200 ppm to determine PFAS toxicity at different chain lengths. The C_lignin_@H-TiO_2_ photocatalyst was included in the *Lemna* media at 0.1 %, 0.2 %, and 0.5 % and was directly exposed and indirectly exposed (through supernatant) to *L. minor* to assess the relative safety of the photocatalyst. Sampling for the toxicology analysis was conducted from the photocatalytic degradation experiments, where aliquots were collected at short-term intervals (0, 0.5, 1, 3, 6 h) in LC water and at long-term intervals (0, 3, 6, 9, 12, 15, 16 d) in rainwater. For the toxicology analysis, each degraded sample (collected at the specified time points) was used to expose L. minor for 7 days, with untreated PFAS rainwater serving as the control. The frond number and surface area of L. minor were recorded on Day 0 (before exposure) and Day 7 (after exposure) using ImageJ (NIH, Bethesda, MD). The changes in frond number and surface area were normalized to the media control (set to 100 %). On Day 7, the plants were collected from each dish and homogenized in 1.5 mL of 80 % acetonitrile. The chlorophyll content was extracted after 48 h (4 °C, dark) and measured by UV-Vis spectrophotometry (Shimadzu UV-1800, Kyoto, Japan) at 663 nm.

### 2.4. Statistical analysis

All *L. minor* studies for the PFAS toxicity assessment were conducted in triplicate with more than one colony of *L. minor* in each group. Vehicle controls were included with each experiment. One-way ANOVA and Tukey’s Test post-hoc analysis were used to assess toxicity endpoints associated with frond number, surface area and chlorophyll content between vehicle and treatment groups. The level of significance was set at p ≤ 0.05.

The EC_50_ was calculated by the Origin software using the dose-response function and the formula: $EC_{50}=10^{LOGx^{0}}$. The top asymptote values were fixed at 1.2 for all groups as the default setting.

## 3. LCMS analysis

### 3.1. Quantitation by triple quadrupole mass spectrometry

Filtered PFAS solutions (10 μL) were loaded into a Gemini 3 μm C18 110 Å, LC Column 50 × 2 mm, C18 column (Phenomenex, USA) to separate the compounds. Ammonium acetate aqueous solutions (20 mM, solvent A) and 100 % methanol (solvent B) were used as mobile phases, with a flow rate of 300 μL/min. The LC gradient started with 95 % solvent A and 5 % solvent B, and this ratio was kept until 1.00 min, then solvent B was increased to 100 % until 5.00 min, and the ratio was maintained until 7.00 min. Subsequently, the ratio was changed to 5 % solvent B and 95 % solvent A until 7.10 min and the ratio was maintained until 15.00 min. The mass spectrometer TSQ Quantiva (Thermo Fisher Scientific, San Jose, CA) was operated with a high temperature ESI source in negative mode. The ion source related parameters were: spray voltage: static; negative ion: 3219 V; sheath gas: 38.3 Arb; aux gas: 1.2 Arb; sweep gas: 2.8 Arb; Ion transfer tube temp: 325 °C; vaporizer temp: 50 °C; CID gas: 1.5 m Torr. The calibration solutions were diluted with water to the corresponding concentration. Both calibration solutions and samples included internal standards with a spiked concentration of 5 ppb. The quantification uses a calibration curve with concentrations ranging from 1 ppb to 250 ppb standard solutions. Quantifications that yield lower than 1 ppb concentration will not be reported based on the quantitation standard.

The environmental water matrices that were subjected to the LCMS include: LC water, Tap water, rainwater, wastewater and groundwater. The sampling for the LCMS analysis was conducted on days 0, 3, 6, 9, and 12.

### 3.2. The high-resolution mass spectrometry characterization

Sample analysis was performed using a Thermo Scientific Q Exactive Focus and LC unit (Ultimate 3000 RS). Samples were separated by injecting 5 μL of sample into the Nova-Pak C18 (2.1 ×150 mm; 4.0 μm) column (Waters). The mobile phase consisted of 10 mM ammonium acetate (eluent A) and methanol (eluent B). The flow rate was set at 300 μL/min with the following gradient: 0–3.0 min 10 % B, 3.0–3.1.0 min 10–40 % B, 3.1–26 min 40–90 % B, 26.1–30.0 min held at 10 % B. The Q Exactive Focus ESI source was operated in full MS (100–1000 *m/z*). The mass resolution was tuned to 70000 FWHM at *m/z* 200. The spray voltage was set to 3.3 kV in negative mode, and 3.5 kV in positive mode, and the sheath gas and auxiliary gas flow rates were set to 40 and 10 arbitrary units, respectively. The transfer capillary temperature was held at 320 °C, and the S-Lens RF level was set at 70 V. Exactive Series 2.11 /Xcalibur 4.2.47 software was used for data acquisition and processing. For the high-resolution mass spectrometry semi-quantitation, the calibration curve was established between 0.2 and 375 ng/L (Table S1) for PFDA, PFNA, PFOA, PFHpA,PFHxA, PFPeA, and PFBA. Each concentration of the calibration solution was analyzed three times on the LC-high resolution mass spectrometry. The calibration curve was used to estimate the quantifiable polyfluorinated carboxylic acids. The limit of detection (LOD) for each PFAS molecule was determined experimentally. Signal-to-noise ratios were calculated from the extracted ion chromatograms (EIC), using accurate mass extraction with a mass accuracy of less than 5 ppm. A signal-to-noise ratio of at least 10:1 was required for a signal to be considered detectable (LOD).

The environmental water matrices that were subjected to the high-resolution mass spectrometry analysis include: LC water treated with six hours using the sun simulator and tap water treated with outdoor sun for 12 days.

### 3.3. Kinetic modeling of PFAS photodegradation

A first-order kinetic model was used to evaluate the kinetic mechanism of PFAS photodegradation [23,24]. The kinetic model described by Eqs. 1–4 was fitted to the experimental data:

(1) $$\frac{dC_{8}}{dt}=-k_{8}•C_{8}\#$$

(2) $$\frac{dC_{7}}{dt}=-k_{7}•C_{7}+k_{8}•C_{8}\#$$

(3) $$\frac{dC_{6}}{dt}=-k_{6}•C_{6}+k_{7}•C_{7}\#$$

(4) $$\frac{dF}{dt}=2•\left[k_{8}•C_{8}+k_{7}•C_{7}+k_{6}•C_{6}\right]\#$$

Where *C*_8_[*mM*] is the concentration of 8-carbon PFOA, $k_{8}\left[\frac{1}{hr}\right]$ is the first-order degradation rate constant for PFOA, *C*_7_ [*mM*] is the concentration of 7-carbon PFHpA, $k_{7}\left[\frac{1}{hr}\right]$ is the first-order degradation rate constant for PFHpA, *C*_6_[*mM*] is the concentration of 6-carbon PFHxA, $k_{6}\left[\frac{1}{hr}\right]$ is the first-order degradation rate constant for PFHxA, and *F*[*mM*] is the concentration of free fluoride ions. Data augmentation with error-informed interpolation was performed to increase the number of data points available for fitting the first-order kinetic model. First, an “error function” (*f*_*err*_(*t*)) was generated for the runtime of the model by interpolating the standard error magnitude from experimental observations with an Akima spline. Next, an Akima spline was used to interpolate between the data points at each experimental condition, resulting in “interpolated data” (*D*_int_(*t*)). A random value between 0 and 1 and an “error factor” (*a*_*err*_) was then multiplied by the error function value at each interpolated time point, resulting in the “interpolated error” (*E*_int_(*t*)) for each interpolated time point. Finally, the *E*_int_(*t*) for each time point was added to the *D*_int_(*t*), resulting in the augmented data set for model fitting. Equation 5 describes this process mathematically.


$$D(t)=D_{\text{int}}(t)+E_{\text{int}}(t)=D_{\text{int}}(t)+a_{\text{err}}•\text{rand}(0\leq x\leq 1)•f_{\text{err}}(t)$$

## 4. Results and discussion

### 4.1. Photocatalyst and environmental parameters’ impacts on L. minor

**Fig. 1.**
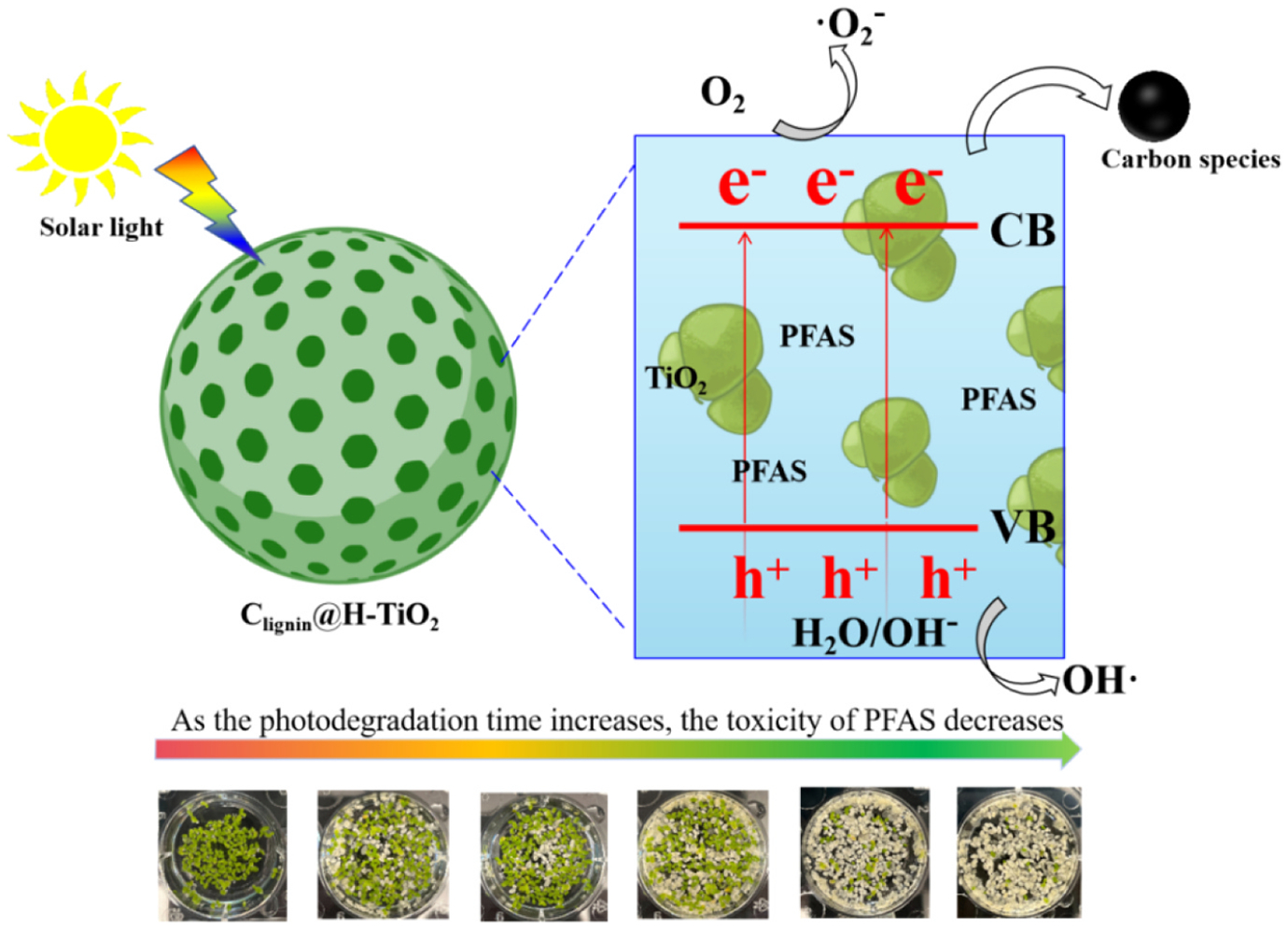
Conceptual design of biomass derived photocatalyst and toxicity assessment using Duckweed as an ecotoxicological model. The photocatalyst is a plant biomass lignin-doped catalyst with a three dimensional spherical structure.

The experimental conceptual design is presented in Fig. 1. The PFOA degraded products and ecotoxicology were evaluated in parallel in a stepwise experimental design. The PFOA was subjected to visible light treatment using the lignin derived photocatalyst in an aqueous solution. The solutions were collected to remove the photocatalyst and used for toxicity assessment. To evaluate the toxicity solely coming from the degraded products, the photocatalyst treated PFOA solution was first filtered through a 0.2 μm filter to remove most of the photocatalyst (average size 0.5 μm [8]). The treated solution was then exposed to *L. minor*.

To test the relative safety of photocatalyst and to ensure negligible impacts from the catalyst on the autotrophic plant species *L. minor*, the photocatalyst was included in the *Lemna* growth media (vehicle control) at 0.1 %, 0.2 %, and 0.5 % with direct contact for 7 days. The results showed negligible impact from direct exposure to 0.1 % photocatalysis in the media for 7 days based on frond number, surface area and chlorophyll content that were similar to vehicle control. However, increasing the inclusion of photocatalysis to 0.2 % and 0.5 % showed significant effect by slowing down the growth and production of chlorophyll over 7 days. For indirect exposure, the photocatalyst was mixed in the media at the same level for 2 h and only the supernatant after centrifugation was exposed to *L. minor*. The impact from indirect exposure of photocatalyst at all 3 inclusion rates on frond number, surface area and chlorophyll content on the endpoint (Day 7) was significantly lowered, compared to the impact of direct exposure on Day 7. Therefore, in our following treatment studies, photocatalyst was included at 0.1 % and removed after degradation treatment is required before disposal.

### 4.2. Evaluation of L. minor response to poly- and perfluorinated carboxylic acids with varying perfluorinated carbon chain lengths

**Fig. 2.**
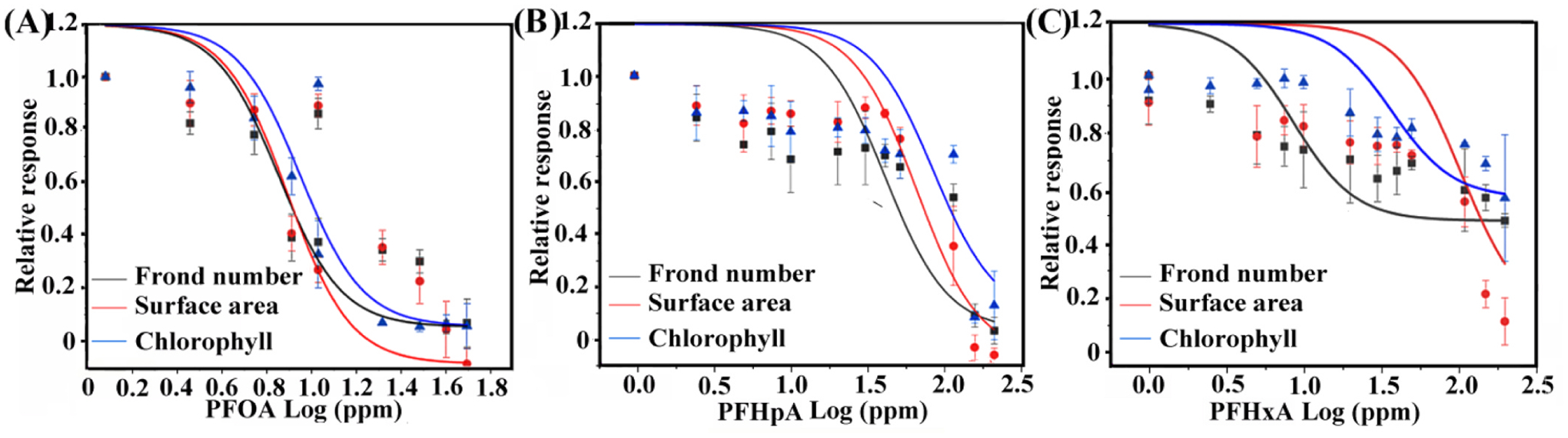
Dose-response curves of the toxicity of PFOA (A) PFHpA (B) PFHxA (C). All values were compared to the vehicle media control, which was adjusted to 100 %. The results are expressed as the average ± standard deviation. The study was independently repeated three times with 2–3 colonies of plants in each group.

To confirm *L. minor’s* sensitivity to PFAS at different chain lengths, we spiked the growth media with PFAS (C8), PFHpA (C7) and PFHxA (C6) at a series of concentrations up to 200 ppm. After 7 days of exposure to *L. minor*, their impact on the growth of frond number and surface area and the production of chlorophyll was compared to the growth media (vehicle control), which was adjusted to 100 %. As shown in Fig. 2, all PFAS dose-dependently decreased *L. minor* status, with the highest concentrations (50 ppm for PFOA and 200 ppm for PFHpA and PFHxA) resulting in 100 % lethality to plants. At these doses, the status of *L. minor*, including frond number, surface area of fronds, and chlorophyll, has reached zero, indicating disintegrated and/or completely bleached plants. Importantly, the longer chain length of PFAS delivered higher toxicity. The EC_50_ values of PFOA were the lowest among other PFAS, showing that lower than 10 ppm PFOA in the media reduced 50 % of frond number, surface area and chlorophyll content. PFHpA was the second most toxic, followed by PFHxA based on EC_50_ (Supplementary Table S2, indicating a positive relationship between chain length and PFAS toxicity on *L. minor*). The chain length-dependent toxicity supports the hypothesis that longer-chain PFAS exert greater phytotoxicity, suggesting that degradation may serve as a viable detoxification strategy. All toxicity parameters using frond number, surface area and chlorophyll content were highly correlated, suggesting high sensitivity and reproducibility of the *L. minor* assay. Based on the EC_50_ of PFOA and PFHpA, the values of 7.5 ppm for PFOA and 40 ppm for PFHpA were chosen as the initial concentrations for the degradation treatments to deliver 50 % reduction for at least one parameter.

### 4.3. Chemical characterization of the photocatalytic degradation of PFOA

**Fig. 3.**
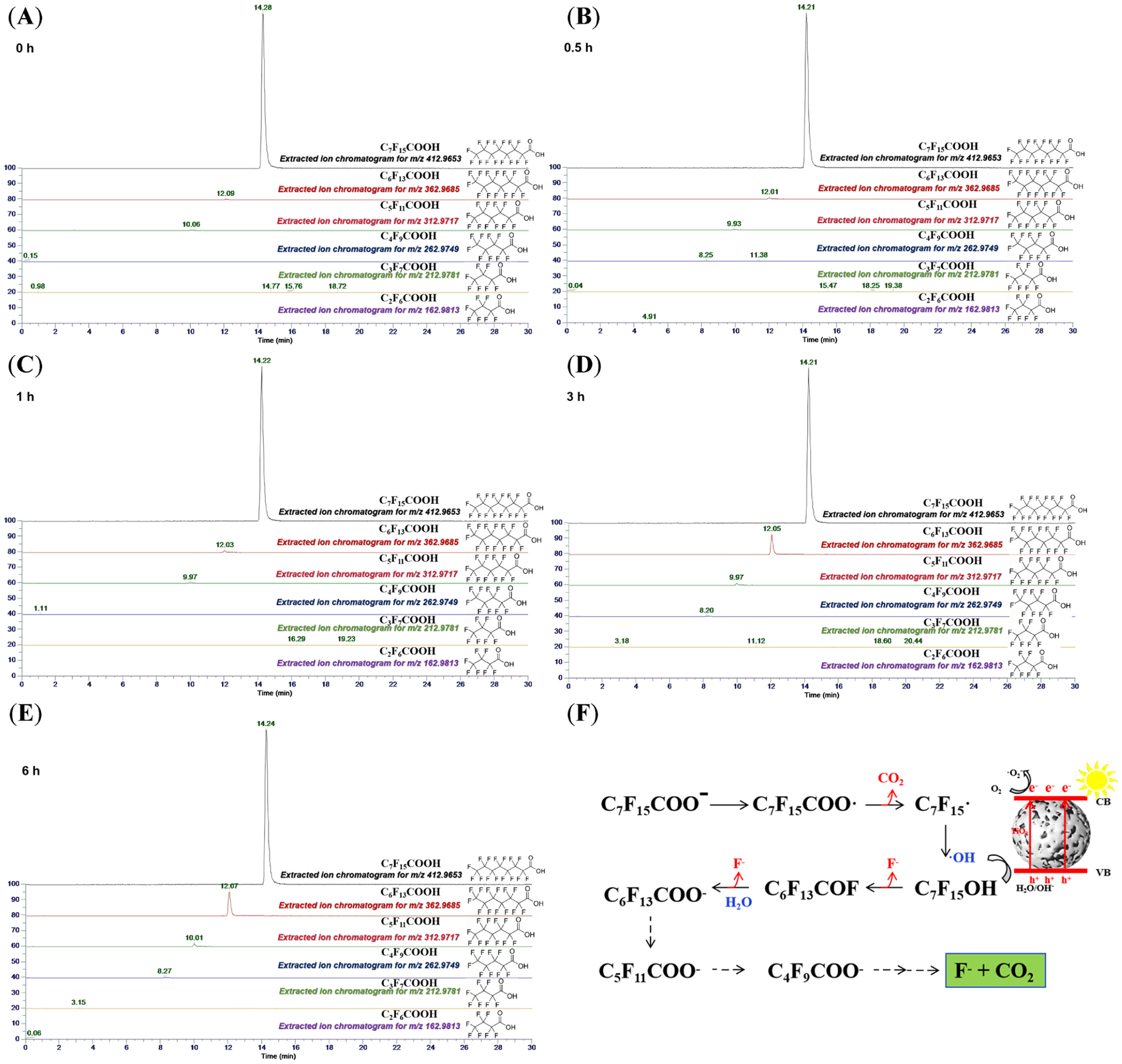
(A-E) The high-resolution mass spectrometry analysis of degradation products from solar light-treated 7.5 ppm PFOA (C_7_F_15_COOH) in LC water and (F) proposed degradation pathways. Individual subfigures are shown separately in Fig. S1.

The degradation behavior of PFOA in LC water and tap water under photocatalysis was further analyzed using high-resolution mass spectrometry (HRMS). To minimize the environmental impurity impacts on the photocatalytic experiments and mass spectrometry detection, the PFOA was prepared using liquid chromatography-mass spectrometry grade water (LC water) as a control. As shown in Fig. 3 (A–E), a progressive generation of shorter-chain perfluorinated carboxylic (PFCAs) acids was observed over time, indicating the efficient catalytic activity of the C_lignin_@H-TiO_2_ photocatalyst. Within six hours visible light treatment, perfluorobutanoic acid (C4) was observed, suggesting the efficient PFOA degradation under visible lights. Initially, the solution contained 7.5 ppm PFOA, which exhibited a significant peak at 14.2 min corresponding to C_7_F_15_COOH. As the reaction proceeded, shorter-chain degradation products, such as PFHpA (C_6_F_13_COOH) and PFHxA (C_5_F_11_COOH), started to appear.

This indicated the stepwise cleavage of C–C bonds within the perfluorinated backbone, which is induced by photocatalysis. The mechanism, shown in Fig. 3 F illustrates the generation of photogenerated electrons (*e*^−^) and holes (*h*^+^) under solar light. The *e*^−^ on the conduction band reacts with adsorbed oxygen to form reactive oxygen species (·O__2__^−^), while the *h*^+^ on the valence band contributes to hydroxyl radical (·OH) formation through water oxidation. These reactive species lead to the successive defluorination and decarboxylation of PFOA, ultimately producing shorter-chain PFCAs and release of fluoride ions (F^−^) and CO_2_ [25,26]. Due to the structure of PFCA molecules, all carbon atoms within PFCA chains (aside from the terminal carbons) are bonded to 2 fluorine atoms. When PFCA chains are degraded one carbon at a time based on the mass spectrometry analysis, meaning if a PFCA molecule of length *n* is degraded into a PFCA of length *n-1*, then it is equivalent to the original PFCA molecule losing one of its non-terminal carbons. Thus, 2 fluorine atoms are released from the PFCA molecule for every single-carbon-atom shortening of the PFCA molecule. At the same time, there could be undetected PFAS degraded products that do not follow the proposed oxidative degradation mechanism. The potential unknown mechanism could significantly contribute to modeling uncertainty and inaccuracy. Nevertheless, the accumulation of PFHpA and PFHxA peaks at 6 h suggests that the degradation pathway proceeded at the carboxyl terminal group, followed by repeated perfluoroalkyl chain shortening. The stability of the shorter-chain PFCAs indicates that further degradation might require extended reaction times or additional oxidative conditions.

The superior performance of the C_lignin_@H-TiO_2_ can be attributed to its enhanced light absorption and charge carrier separation by carbon, as evidenced by the effective mineralization of PFOA and the formation of intermediates. The incorporation of carbon likely reduces the recombination of photogenerated *e*^−^*/h*^+^ pairs and improves surface reactivity, thereby accelerating the degradation process [8]. This observation aligns with the proposed mechanism, where the photocatalytic activity is maintained throughout the reaction, leading to sustained PFCA formation and eventual mineralization into non-toxic end products.

The degradation process of PFOA (C_7_F_15_COOH) and PFHpA (C_6_F_13_COOH) under photocatalysis was further supported by the time-dependent peak area changes in the high-resolution mass spectrometry data. As shown in Supplementary Table S3, at 0 h, only the initial PFOA (7.5 ppm) and PFHpA (40 ppm) were detected, with no shorter-chain PFAS products present. However, shorter-chain PFAS began to emerge as the reaction progressed, including PFHxA (C_5_F_11_COOH), PFPeA (C_4_F_9_COOH), and PFBA (C_3_F_7_COOH), with their peak areas increasing over time. As the reaction progressed, the peak area of PFOA gradually decreased, dropping to 5.47 × 10^9^ after 6 h of solar light treatment, indicating partial degradation. In the meantime, shorter-chain intermediates appeared, such as PFHpA (C_6_F_13_COOH) and PFHxA (C_5_F_11_COOH). The PFHpA peak area increased from 3.6 × 10^7^ at 0.5 h to 6.6 × 10^8^ at 6 h, suggesting its formation as a primary degradation intermediate. Similarly, PFHxA showed a progressive increase, reaching 6.12 × 10^7^ at 6 h.

For the PFHpA solution (40 ppm), the degradation process followed a similar trend, though PFHpA exhibited higher stability compared to PFOA. Its peak area declined only slightly from 1.1 × 10^10^ at 0 h to 9.76 × 10^9^ at 6 h. The shorter-chain intermediates, including PFHxA (C_5_F_11_COOH, 3.64 ×10^7^ at 0.5 h to 1.15 ×10^9^ at 6 h) and PFPeA (C_4_F_9_COOH, 6.23 ×10^5^ at 0.5 h to 8.27 ×10^7^ at 6 h), increased over time, further confirming the stepwise breakdown of the perfluorinated carbon chains. The emergence of PFPeA and PFBA (C_3_F_7_COOH) in both solutions at later stages underscores the sequential defluorination and decarboxylation processes. Even though the PFBA peaks are present in the high-resolution mass spectra, the PFBA concentration is around the detection limit. Nevertheless, we consistently observed the PFBA degradation products in the oxidative degradation process, which was detected in the PFOA degradation sample at 6 h (peak area at 4.32 × 10^5^) and in the PFHpA sample as early as 1 h (5.05 × 10^5^), reaching 1.09 × 10^7^ at 6 h. This highlights the sustained catalytic activity of C_lignin_@H-TiO_2_, which can progressively break down even relatively stable intermediates and ultimately achieve less toxic end products.

**Fig. 4.**
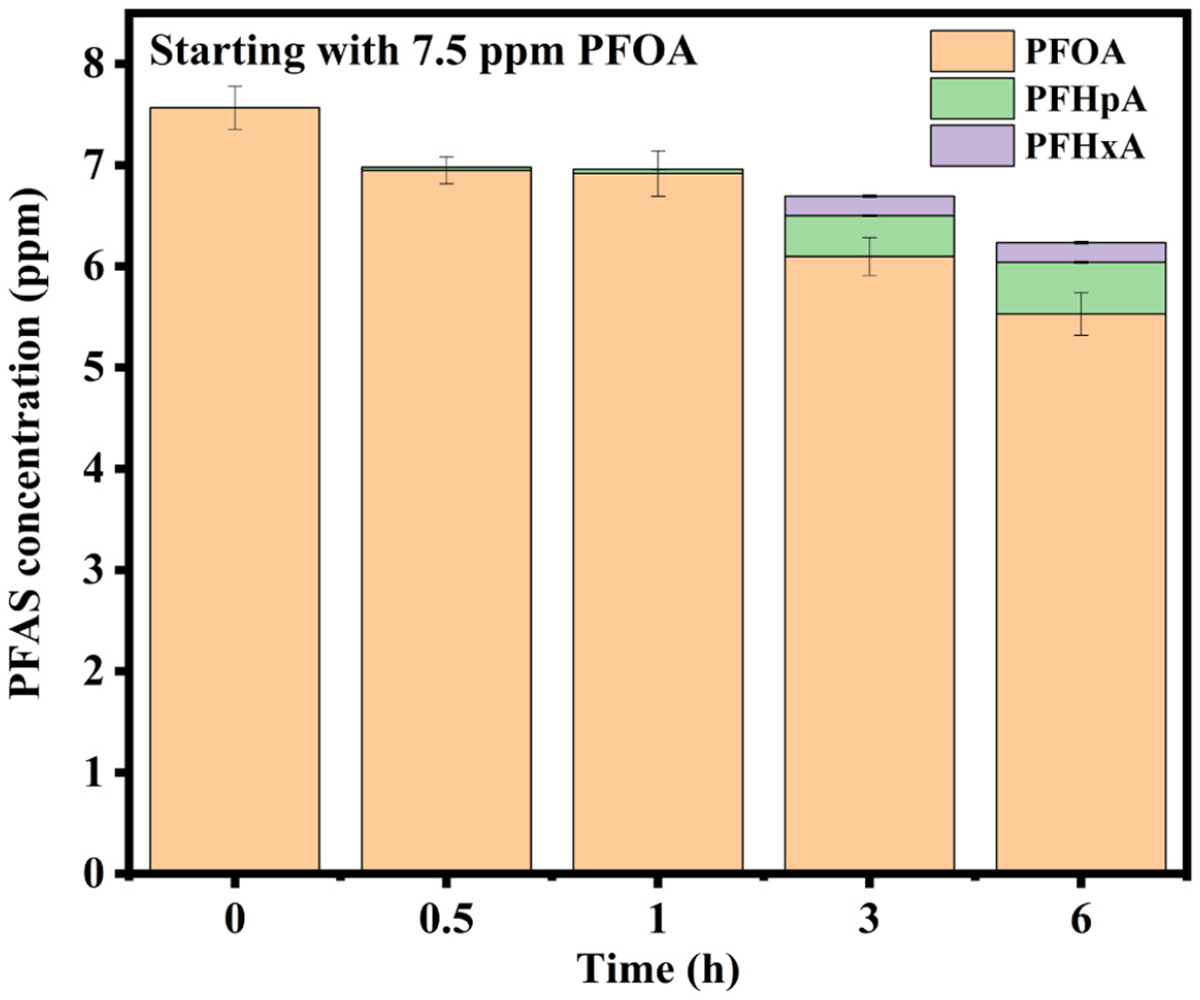
The LC-triple quadrupole mass spectrometry analysis of degradation products from solar light-treated 7.5 ppm PFOA (C_7_F_15_COOH) in LC water for 6 h.

To quantify all PFOA and PFHpA degradation species, quantitation by LC-triple quadrupole mass spectrometry analysis was conducted on the degradation products from solar light-treated 7.5 ppm PFOA and 40 ppm PFHpA in LC water over 6 h, as shown in Fig. 4 and Table S4. The concentration of PFOA gradually decreases about 2 ppm over 6 h, with a significant drop observed between 3 and 6 h. Concurrently, the formation of shorter-chain transformation products, PFHpA and PFHxA, becomes evident, suggesting stepwise defluorination and chain-shortening reactions. This indicates that PFOA undergoes partial degradation and generates measurable quantities of intermediates. The LC water containing 7.5 ppm PFOA in the absence of photocatalyst was subject to the sun-light treatment as a control (Table S5). No quantifiable degraded products (i.e., PFPeA, PFHxA, PFPeA) were observed.

### 4.4. Unexpected PFAS detected in texas potable water

**Fig. 5.**
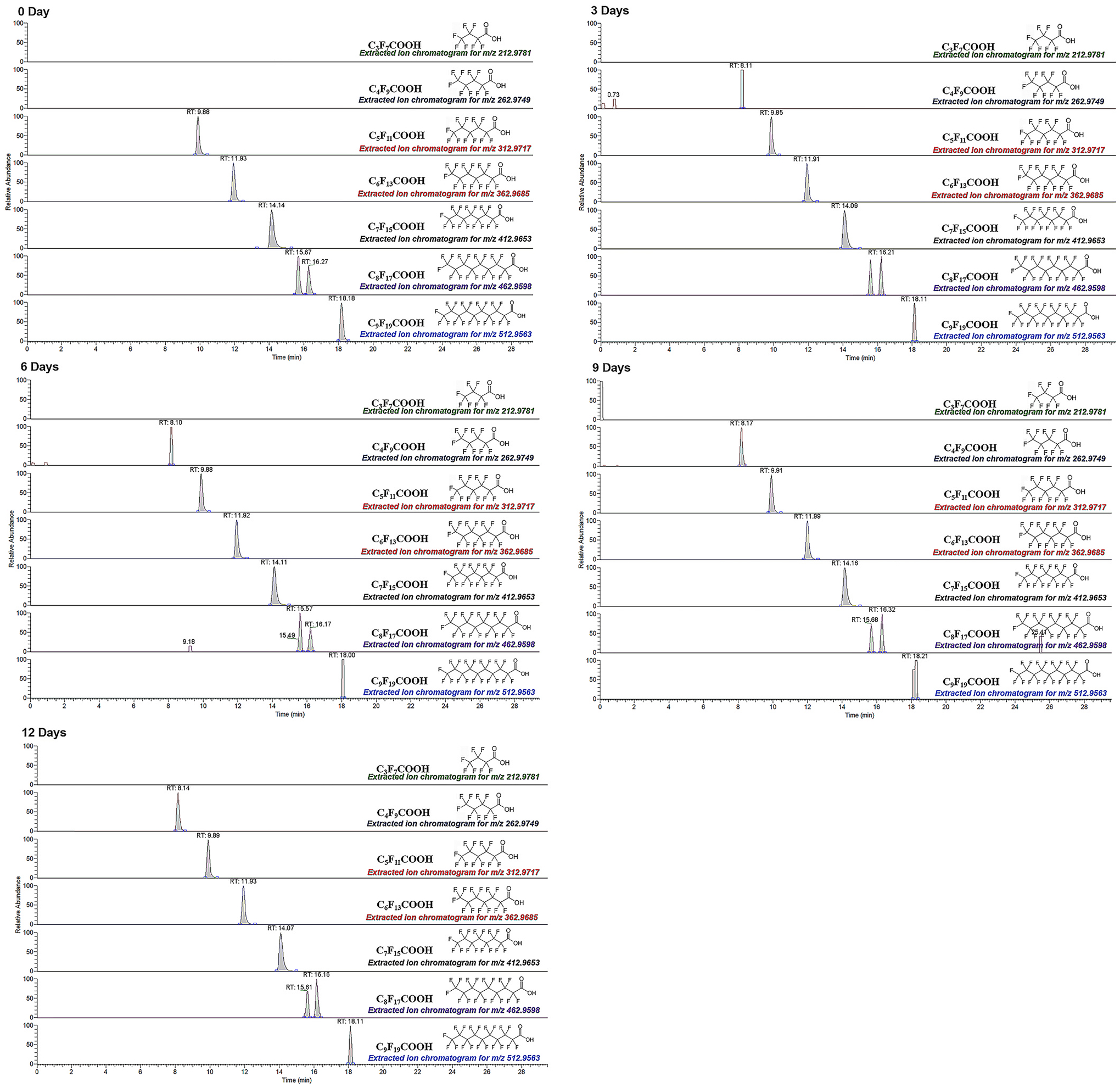
The high-resolution mass spectrometry analysis of degradation products from natural light-treated 7.5 C_7_F_15_COOH in tap water. Individual subfigures are shown separately in Fig. S2.

Interestingly, the potable tap water used already contained PFAS molecules such as C_9_F_19_COOH (PFDA) and C_8_F_17_COOH (PFNA), which were not expected. The tap water PFAS contamination was discovered because both the LC water and tap water were spiked with 7.5 ppm PFOA to perform the catalysis experiments. In the high-resolution mass spectrometry analysis, the peaks of PFDA and PFNA showed up in the tap water spiked with PFOA, but not in the LC water spiked with PFOA. Due to the semi-quantitative analysis nature of the high-resolution mass spectrometry analysis, the concentrations of PFDA and PFNA were estimated based on the peak areas measured in the LC-high resolution mass spectrometry analysis. Using the calibration established by the LC-high-resolution mass spectrometry analysis, the concentration of PFNA is estimated to be around 1 ppb, close to the detection limit (Table S1). The PFDA is below the quantitation limit. The estimated concentrations of PFNA and PFDA are marked by red dots in the calibration curve. As a matter of fact, when the tap water spiked with PFOA was subjected to high-resolution mass spectrometry (day 0 sample, Fig. 5), several PFAS species, including PFDA, PFNA, PFHpA, and PFHxA, were detected. This observation confirms that tap water is potentially contaminated by various PFAS, highlighting the broad PFAS presence in the environment. With increasing sunlight exposure, significant changes in the PFAS peak areas were observed. By day 3, a new peak corresponding to PFPeA appeared, indicating the initial breakdown of longer-chain PFAS into shorter-chain products. This trend continued as the exposure time increased, with the peak area for PFPeA progressively increasing over time, particularly by day 12. Conversely, the peak area for PFDA steadily decreased, suggesting the effective photodegradation of long-chain PFAS into shorter-chain species. These results demonstrate that natural sunlight can effectively degrade PFAS, particularly longer-chain species, into shorter-chain compounds. Meanwhile, the initial PFAS mixture could complicate the degradation dynamics. Shorter-chain PFAS are generally considered to be less toxic and more mobile in aquatic environments, thereby reducing the overall toxicity of the contaminated water. Additionally, the formation of intermediate products such as PFPeA during the degradation process indicates stepwise breakdown pathways under natural light conditions. The gradual reduction in longer-chain PFAS concentrations, coupled with the accumulation of shorter-chain intermediates, underscores the importance of prolonged sunlight exposure for efficient degradation. This finding aligns well with the previously discussed results, further supporting the potential of sunlight-driven photodegradation as an environmentally friendly approach to mitigate PFAS toxicity and pollution.

The changes in peak areas of degradation products were also evaluated by high-resolution mass spectrometry as shown in Table 1. On day 0, in addition to the primary spiked compound (C_7_F_15_COOH), several long-chain PFAS, including PFDA, PFNA, PFHpA, and PFHxA were detected. This finding suggests pre-existing PFAS contamination, consistent with earlier results. Over time, the degradation of these long-chain PFAS was observed, accompanied by the emergence of shorter-chain products. The presence of C_4_F_9_COOH (PFBA) was first observed on day 3, with a sharp increase in its peak area by day 12. These trends indicate that natural sunlight effectively breaks down longer-chain PFAS into shorter-chain compounds over time, reducing the overall carbon chain length and potentially decreasing toxicity. It is evident that the degradation rate for 12-days treatment (Fig. 5) is slower than the 6-hours treatment (Fig. 3), as the appearance of PFPeA showed up by day 3, but was already detected within 6 h. It is likely due to the fact that the 12-day treatment was performed in a closed plastic bottle, which was shielded from the natural light radiation. The six-hour treatment experiments were performed with direct exposure to the lab light source. Interestingly, we observed two peaks of PFNA, which could be due to the isomers of PFNA. Indeed, linear and branched PFAS such PFOS have been reported before [27]. Thus, the PFNA could also have isomers with linear and branched structures, and the same molecular weight. Regardless of the presence of unexpected PFAS in the water matrices, we do not expect the low concentration of PFAS contaminants to interfere with our result interpretation for two reasons: 1) the PFAS contaminants’ concentrations are much lower than our spiked PFOA concentration; 2) the PFAS contaminants, such as the longer chain PFDA and PFNA molecules, go through oxidative degradation, and the entire PFAS mixture is evaluated by the toxicity evaluation for the combined toxicity.

In the tap water, the original PFDA and PFNA concentration is minimum, which is close to the detection limit. Accordingly, the PFDA and PFNA were degraded by the photocatalyst as the peak area kept decreasing over the six-hour natural sunlight treatment (Table 1). Meanwhile, the spiked PFOA at the 7.5 ppm concentration was degraded and decreased significantly over time. This dynamic indicates a continuous degradation pathway for the extended exposure compared to the six-hour treatment, where longer-chain PFASs break down into intermediate compounds, including PFOA, before further transforming into shorter-chain PFAS. The gradual increase in the peak areas of shorter-chain PFAS (i.e., PFPeA begins to show up in the day 3 sample, and the peak area increases over time) in the first six days further supports this sequential degradation mechanism (Table 1), which also suggests that sunlight not only facilitates the breakdown of longer-chain PFAS but also drives the progressive formation and accumulation of shorter-chain intermediates. The degradation trend is also consistent with the short time (i.e., 6 h) irrigation treatment (Table S3). This trend highlights the persistence of PFAS transformation under natural light conditions in the extended sunlight treatment and underscores the importance of understanding these pathways to fully assess the environmental impact and toxicity of PFAS degradation products. These findings are consistent with the overall trend observed in the lab bench test 6-hour degradation experiments, where natural light catalyzed the breakdown of PFAS into shorter and less toxic intermediates. However, after day six, the concentrations of longer carbon chain PFAS (I.E., C9, C8) seem to become stabilized. The middle carbon chain length PFAS concentrations (i.e., C7 and C6) have marginal increases after six days. On the other hand, the shorter chain PFAS (i.e., C5) increased more significantly. The data highlights the potential of sunlight as an ecofriendly and sustainable approach for PFAS remediation while also emphasizing the need for prolonged exposure or additional treatment strategies to achieve more efficient mineralization and minimize residual contamination.

### 4.5. Kinetic models for PFOA and its degradation products

First-order and pseudo-first-order kinetic models have been used in previous studies to model photodegradation of PFAS [28–30]. However, most previous studies focused on assessing photodegradation mechanisms in solution samples with minimum environmental interference. The present study focuses on PFAS photodegradation in realistic, complex environmental samples, such as wastewater. Thus, the degradation mechanisms may be different from those in samples with fully defined compositions. First-order kinetic models fit to time-course concentration data for the natural light PFAS photodegradation experiments, and the goodness-of-fit (R^2^ and estimated parameter confidence intervals) are used to evaluate how well first-order kinetics can describe PFAS photodegradation in realistic samples.

**Fig. 6.**
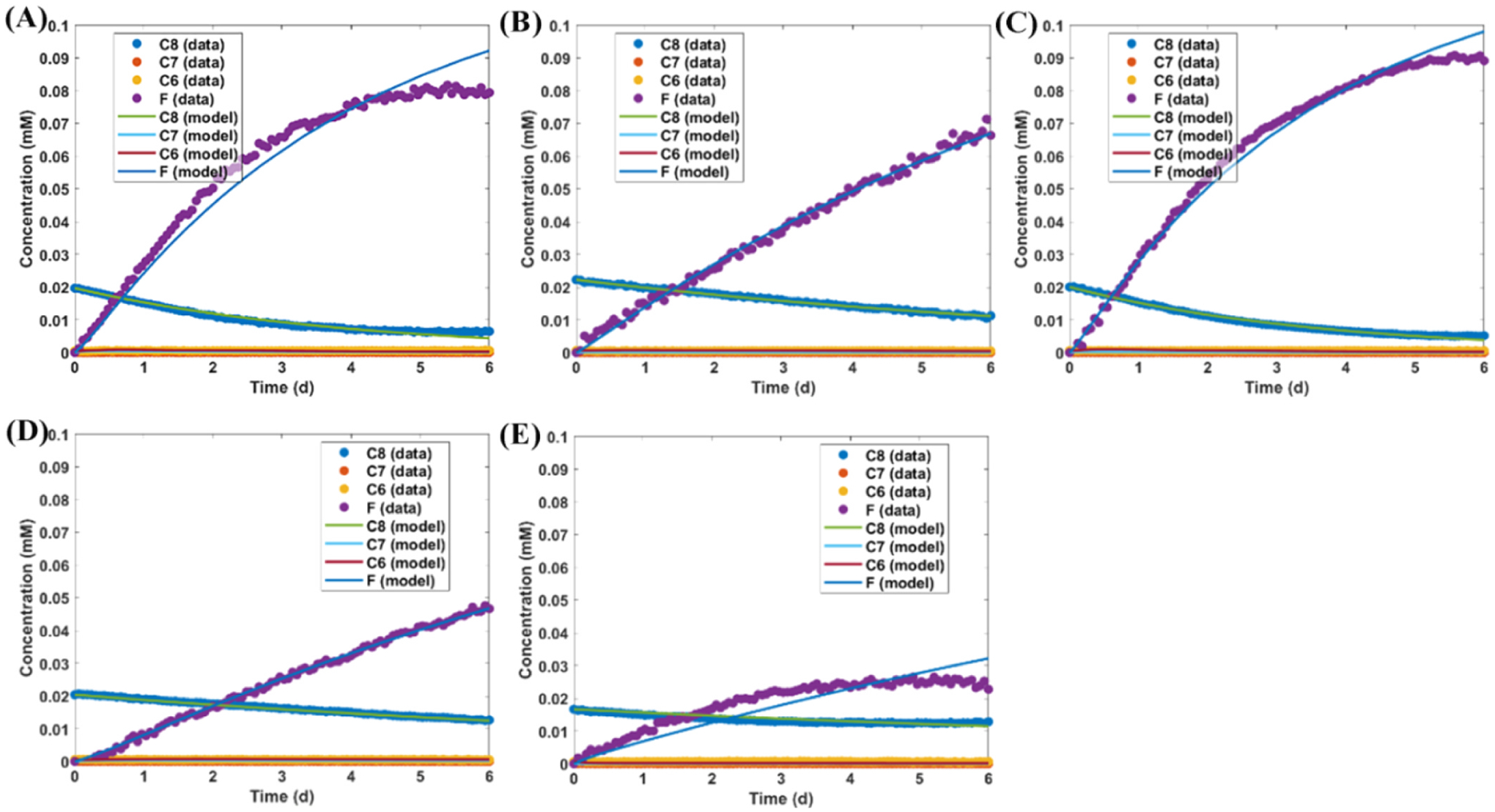
Fitting results of first-order photodegradation kinetic model to the error-informed interpolated dataset for (A) LC water, (B) tap water, (C) rainwater, (D) groundwater, and (E) wastewater.

In our modeling fitting, the first-order kinetic model fits better than the pseudo-first-order kinetic models, even though the first-order fitting does not fit for all species. As a matter of fact, PFOS and F^−^ present reasonably fitting, but the C7/C6 species do not fit well. (Fig. 6 and Table S6 and S7). Tap water, rainwater, and groundwater fit better than LC water and wastewater. Particularly, the wastewater sample, which is likely the most complex of all observed samples, has the lowest quality fit to the first-order photodegradation model. Table S6 summarizes the 95 % confidence intervals (CIs) for the parameter predictions in the first-order model for each observed sample; parameter CIs that include 0 indicate that there is insufficient confidence that the parameter is necessary for the model, suggesting the model is not well-suited to describe the experimental data. The wastewater sample is the only sample that was unable to have all the parameters necessary when fitting a first-order model. Table S7 summarizes the coefficient of determination (R^2^) values for the data trends in each sample. For the species measured in the experiment, PFOA concentration fits well into the first-order kinetics model, with the PFHpA and PFHxA fitting being poor for all samples. However, for wastewater, the PFOA and F^−^ fitting are significantly lower quality than in other samples. The goodness-of-fit analysis suggests the photodegradation mechanism in the wastewater sample is the most different from first-order kinetics out of all of the samples that were tested.

Furthermore, data points for the fluoride ion time-course concentration were calculated via mass balance from measured PFOA, PFHpA, and PFHxA time-course concentrations and subjected to the same data augmentation as other samples as described in “Methods”; thus, it represents all fluorine atoms released from PFAS molecules regardless of whether they remain as free ions or get incorporated into other chemical species. It is assumed that for every instance where a PFAS chain is shortened by 1 carbon atom, 2 fluoride ions are released from that PFAS chain as well. The calculated F^−^ time-course data was included in the regression to fit the first-order model. Furthermore, the PFAS photodegradation kinetics stagnated for most samples after 6 days, likely due to each sample being run in a closed system; the first-order model fitting was only performed on the first 6 days of observations for each sample.

### 4.6. Correlation of PFOA photocatalytic degradation and detoxification in environmental matrices

**Fig. 7.**
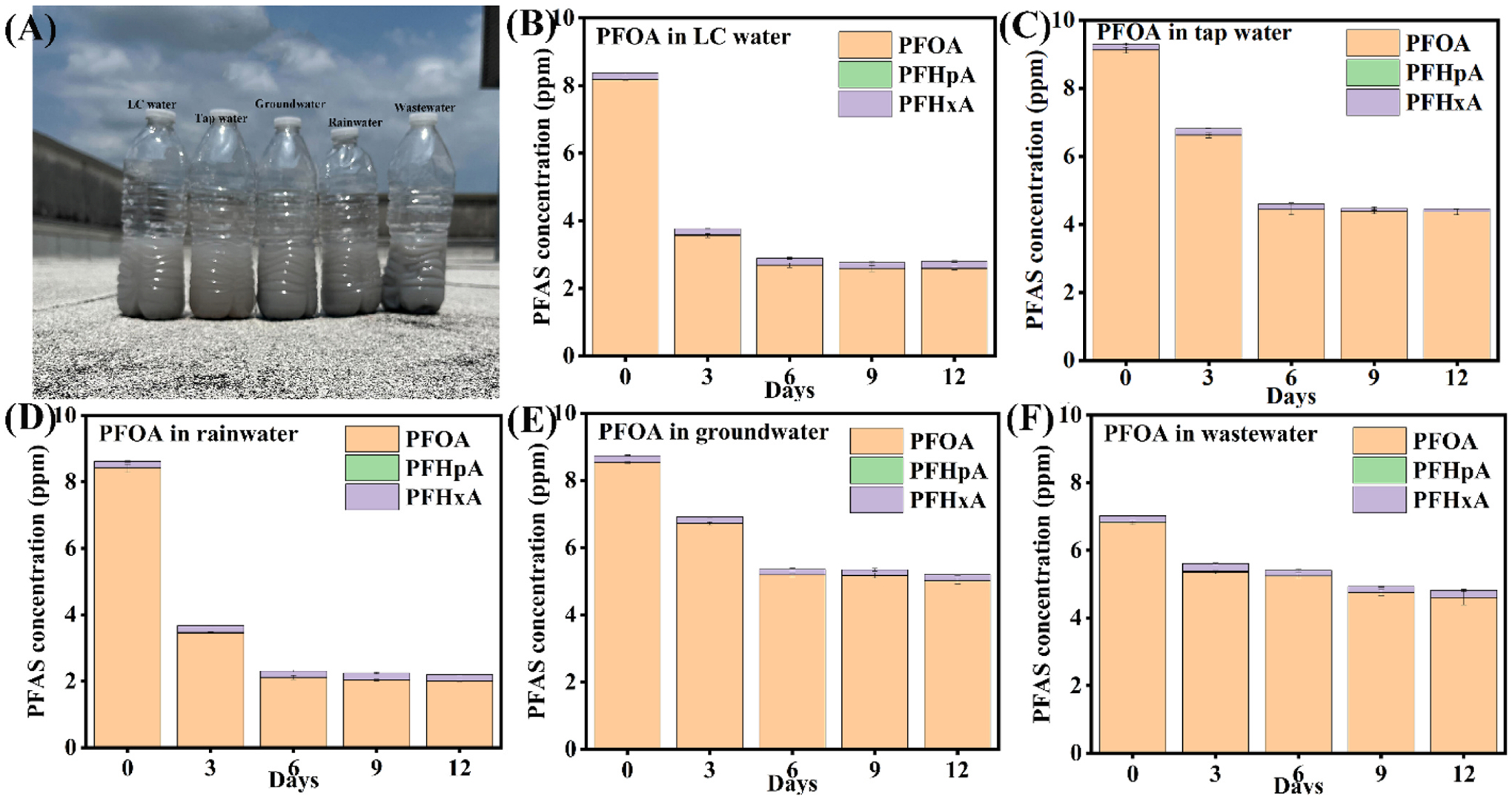
(A) Photodegradation of PFOA under natural sunlight in different aqueous matrices. The picture captures the treatment environment on the top of a research building at the Texas A&M University campus. PFOA degradation under natural sunlight in (B) laboratory-grade (LC) water, (C) tap water, (D) rainwater, (E) groundwater, and (F) wastewater.

To evaluate the PFOA degradation under sunlight with more environmentally relevant conditions, 7.5 ppm PFOA was spiked into various water matrices (i.e., groundwater, rainwater, wastewater, lab tap water, using the LC water as a control) in plastic drinking water bottles. The bottles were subjected to sunlight for a 16-day period (including nights). One sample was taken from each bottle every three days for 12 days and subjected to LC-MS analysis and toxicological assessment (7 samples taken at day 0, 3, 6, 9, 12, 15 and day 16). Fig. 7 A illustrates the experimental setup, showing PFOA solutions in different water matrices in PET plastic bottles exposed to sunlight. Fig. 7 B–F shows plots of the concentrations of different PFAS in the photocatalytic reactive system.

Figures (B) to (F) provide insights into PFOA degradation in different water matrices, including LC water, tap water, rainwater, groundwater, and wastewater. In all samples, a consistent trend of decreasing PFOA concentration was observed with increasing exposure time. The presence of PFHpA was detected on day 0. The observation of PFHpA at the start suggests that it may originate from environmental contamination. The rate of PFOA degradation varied among the water matrices, influenced by their unique compositions. LC water exhibited a rapid decline in PFOA concentration, potentially due to its relatively pure composition that minimizes competing reactions (Fig. 7B). Tap water and rainwater showed similar trends (Fig. 7C–D), with PFHpA formation becoming higher with light treatment. Groundwater and wastewater (Fig. 7E–F) with their higher organic and inorganic content, demonstrated slightly slower degradation rates, suggesting that matrix complexity may impede catalytic efficiency. The results highlight the potential of sunlight-driven photocatalysis as an effective and versatile method for degrading PFOA in various water matrices. Its consistent efficiency in reducing PFOA levels and toxicity demonstrates its adaptability to different environmental conditions. The significant reduction in toxicity observed in real-world samples further confirms its practicality for mitigating PFOA contamination in environmental applications.

**Fig. 8.**
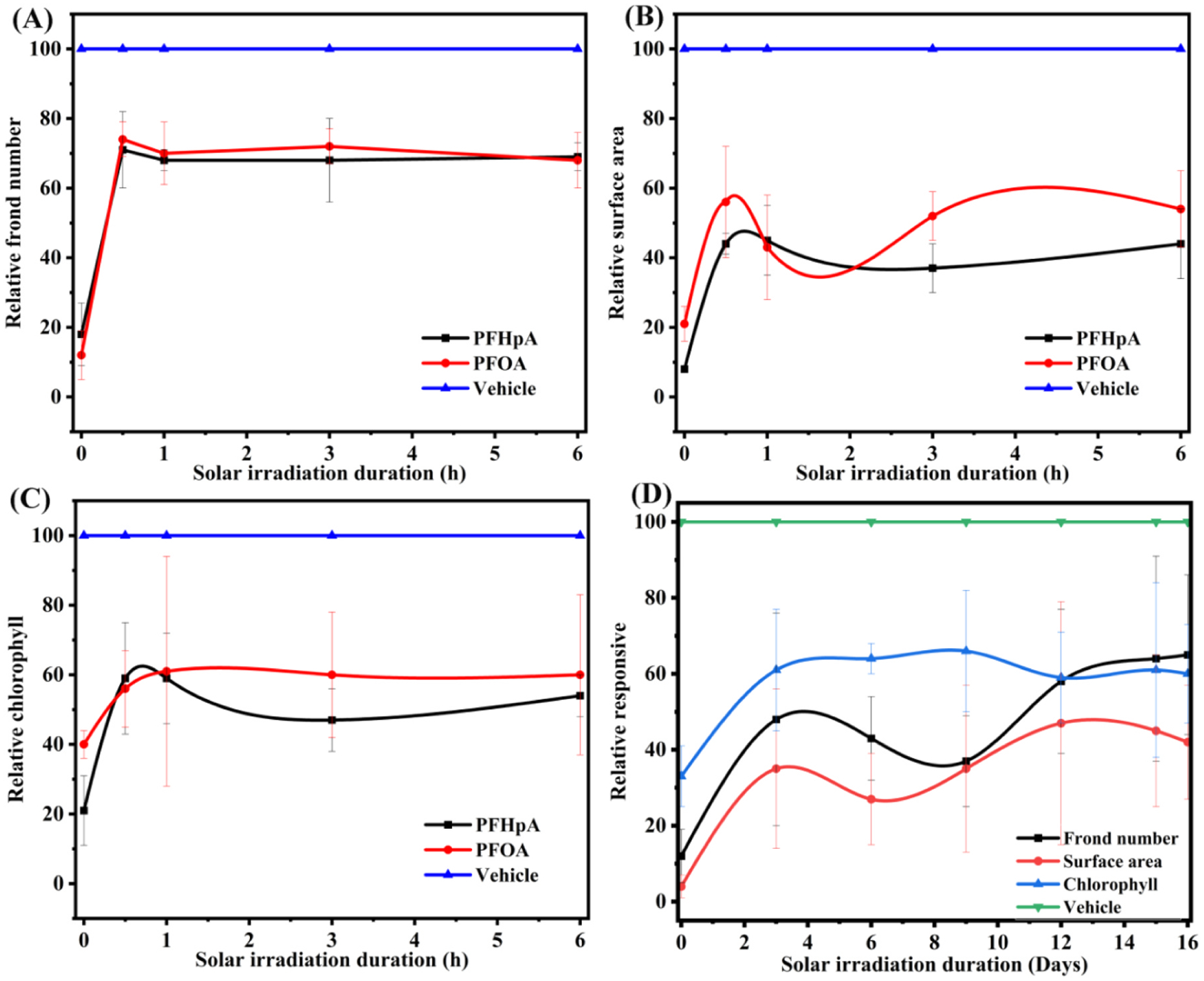
Frond number (A) surface area (B) chlorophyll (C) of *L. minor* following short-term (A-C) and long-term (D) treatments. All values were compared to vehicle media control, which was adjusted to 100 %. (A) (B) and (C) are experimental results using a sun simulator as the light source. (D) is the 16-day experimental results using capped plastic water bottles as containers and outdoor sunlight as the light source. In all figures, time 0 means the PFAS solution has not been subject to light treatment so no degradation would be expected.

For short-term degradation treatments, LC water was spiked with 7.5 ppm PFOA or 40 ppm PFHpA based on EC_50_ values and irradiated by a solar simulator equipped with a xenon lamp for 0, 0.5, 1, 3, and 6 h. Treated water samples were exposed to *L. minor* for 7 days and toxicity endpoints were compared with the growth media as the vehicle control. At the starting point (0 h), both PFOA and PFHpA exhibited toxicity that resulted in approximately 20 % growth in frond number, 10–20 % growth in surface area and 20–40 % production of chlorophyll, compared to vehicle media control, which was adjusted to 100 % (Fig. 8A–C). However, this toxicity was reduced following sunlight irradiation, where all 3 parameters were improved. Specifically, the relative growth of the frond number increased to 70 %, the relative surface area increased to 50 %, and the relative chlorophyll content increased to 60 % following degradation treatments. Importantly, the detoxification following sunlight irradiation was consistent and reached a plateau, suggesting a stable and persistent degradation reaction between 0.5 and 6 h. Additionally, the reaction occurred fast, and the detoxification reached a plateau within 0.5 h of contact. These findings suggest that short-term degradation using a solar simulator effectively reduced PFOA and PFHpA toxicity to *L. minor* and the reaction was fast and stable for up to 6 h.

As rainwater matrices are most likely to interact with ecosystem species, such as aquatic species duckweed, rainwater matrices were selected for the ecotoxicity evaluation. The ecotoxicity evaluation for 7.5 ppm PFOA in rainwater was studied with longer treatment durations for 0, 3, 6, 9, 12, 15, and 16 days in a plastic bottle under natural sunlight. The plastic bottle had a shading impact on the sunlight, and the natural sunlight is likely less intense than the sun simulator. The long-term treatments (for days) had similar trends to the short-term treatments (hours), where relative growth as determined by frond number, surface area, and chlorophyll content improved from 10 %, 5 %, and 30 % on Day 0–65 %, 42 %, and 60 % on Day 16, respectively (Fig. 8D). However, contrasted to the stable detoxification with short-term treatment, the detoxification continued during prolonged treatment. Specifically, the frond number and surface area continued to rise from 48 % and 35 % on Day 3 to higher levels by Day 16. This result suggested that the degradation reaction persisted throughout the 16-day treatment period, and potentially could result in even higher detoxification with longer treatment durations.

It is also interesting to observe that the toxicity decrease seems to be most significant at the beginning of the treatment. For example, for the six-hour treatment, the toxic impact on fond numbers, surface area, and chlorophyll measurement is more comparable for the 0.5, 1, 3, and 6-hour treatment samples. On the contract, the toxicity impact is more significant for the non-treated sample (i.e., 0 h) compared to the treated samples (Fig. 8A–C). A similar trend is observed for the extended treatment samples, where the toxicity decrease is most significant, as shown in the day 3 treatment sample (Fig. 8D). The most significant toxicity drop in the early stage of the treatment is likely due to: 1) PFOA is more toxic than the shorter-chain PFAS such as PFHpA and PFHxA (Table S2, EC_50_ comparison of PFOA, PFHpA, and PFHxA), which leads to the fact that detoxification is more significantly associated with the longer-chain PFOA concentration; 2) the significant PFOA removal in the entire PFAS mixtures from the onset (Fig. 4 and Fig. 5). This observation suggests the importance of destroying the longer chain PFAS molecules into less toxic shorter chain PFAS. Meanwhile, the PFOA degradation is indeed a complex process, which involves the disappearance of PFOA and increasing concentrations of short-chain PFAS such as PFHpA to PFBA. The mass spectrometry quantification can give an account of measurable PFAS presence. The toxicity evaluation reflects the impacts of all PFAS species. Thus, a system evaluation of toxicity in combination with PFAS species measurement by mass spectrometry shed more light on the PFAS degradation remediation process.

## 5. Conclusions

In this study, we systematically evaluated PFOA degradation in various environmental water matrices and correlated the degradation and proof-of-concept *in vivo*. This was accomplished with a living organism (*Lemna minor*) that has been extensively used to monitor water quality and to predict the toxicity of hazardous pollutants and toxic chemicals in the environment. The environmental matrix interference had different impacts on PFOA degradation in the solution phase, with wastewater having the most profound impact on the degradation kinetics. We show that visible-light-driven photocatalysis is a viable way to degrade PFOA in the natural environment, with the significant benefit of minimizing PFAS toxicity to the ecosystem. Particularly, prolonged photocatalytic treatment led to continuous detoxification, which was not observed in the short-time treatment system (i.e., six hours). Our study not only highlights the feasibility and benefit of photocatalytic remediation of PFAS in the natural environment but also suggests that potential novel detoxification pathways exist in the ecosystem and warrant further study.

## Supplementary Material

Supplementary material 2

Supplementary material 1

## Figures and Tables

**Table 1:** Peak area comparison of degradation products from natural light-treated tap water spiked with 7.5 ppm PFOA (C_7_F_15_COOH).

Sample	7.5 C_7_F_15_COOH (area)				
	0 day	3 days	6 days	9 days	12 days
C_9_F_19_COOH (PFDA)	5.5 × 10^6^	4.85 × 10^4^	1.47 × 10^4^	2.52 × 10^4^	1.01 × 10^5^
C_8_F_17_COOH (PFNA)	3.46 × 10^6^	2.92 × 10^5^	1.52 × 10^5^	2.43 × 10^5^	2.43 × 10^5^
C_7_F_15_COOH (PFOA)	1.49 × 10^10^	7.66 × 10^9^	6.78 × 10^9^	6.79 × 10^9^	7.53 × 10^9^
C_6_F_13_COOH (PFHpA)	2.74 × 10^7^	2.7 × 10^7^	3.39 × 10^7^	4.57 × 10^7^	5.99 × 10^7^
C_5_F_11_COOH (PFHxA)	6.85 × 10^7^	6.31 × 10^7^	5.93 × 10^7^	6.19 × 10^7^	6.21 × 10^7^
C_4_F_9_COOH (PFPeA)	-	2.09 × 10^4^	1.47 × 10^4^	2.14 × 10^5^	8.67 × 10^5^
C_3_F_7_COOH (PFBA)	-	-	-	-	-

## Data Availability

Data will be made available on request.

## Appendix A. Supporting information

Supplementary data associated with this article can be found in the online version at doi:10.1016/j.hazmo.2026.100016.

## References

[1] Dickman RA, Aga DS, A review of recent studies on toxicity, sequestration, and degradation of per-and polyfluoroalkyl substances (PFAS), J. Hazard Mater 436 (2022) 129120, 129120. 10.1016/j.jhazmat.2022.129120 PMC12981655 35643010

[2] Domingo JL, Nadal M, Human exposure to per-and polyfluoroalkyl substances (PFAS) through drinking water: a review of the recent scientific literature, Environ. Res 177 (2019) 08648. 10.1016/j.envres.2019.108648 31421451

[3] Lenka SP, Kah M, Padhye LP, A review of the occurrence, transformation, and removal of poly-and perfluoroalkyl substances (PFAS) in wastewater treatment plants, Water Res. 199 (2021) 117187. 34010737 10.1016/j.watres.2021.117187

[4] Vierke L, Staude C, Biegel-Engler A, Drost W, Schulte C, Perfluorooctanoic acid (PFOA)—main concerns and regulatory developments in Europe from an environmental point of view, Environ. Sci. Eur 24 (2012) 1–11.

[5] Leung SCE, Shukla P, Chen D, Eftekhari E, An H, Zare F, Ghasemi N, Zhang DK, Nguyen N-T, Li Q, Emerging technologies for PFOS/PFOA degradation and removal: a review, Sci. Total Environ 827 (2022) 153669. 35217058 10.1016/j.scitotenv.2022.153669

[6] Cao C-S, Wang JZ, Yang LP, Wang JW, Zhang YQ, Zhu LY, A review on the advancement in photocatalytic degradation of poly/perfluoroalkyl substances in water: Insights into the mechanisms and structure-function relationship, Sci. Total Environ 946 (2024) 174137, 174137. 10.1016/j.scitotenv.2024.174137 38909806

[7] Alalm MG and Boffito DC, Mechanisms and pathways of PFAS degradation by advancedoxidation and reduction processes: A critical review. Chem Eng J 450, 138352.

[8] Zhang W, Liang YH, Hu C, Li WW, Lai JR, Chen KN, Xiang SS, Niedzwiedzki D, Wu J, Li A, 3D structure-functional design of a biomass-derived photocatalyst for antimicrobial efficacy and chemical degradation under ambient conditions, Green. Chem 26 (2024) 10139–10151. 39247131 10.1039/d4gc01246a PMC11373602

[9] Amstutz VH, Cengo A, Gehres F, Sijm DTHM, Vrolijk MF, Investigating the cytotoxicity of per-and polyfluoroalkyl substances in HepG_2_ cells: a structure-activity relationship approach, Toxicology 480 (2022) 153312. 36075290 10.1016/j.tox.2022.153312

[10] Sodani K, Ter Braak B, Hartvelt S, Boelens M, Jamalpoor A, Mukhi S, Toxicological mode-of-action and developmental toxicity of different carbon chain length PFAS, Toxicol. Lett 405 (2025) 59–66. 39933616 10.1016/j.toxlet.2025.02.003

[11] Buck RC, Franklin J, Berger U, Conder JM, Cousins IT, De Voogt P, Jensen AA, Kannan K, Mabury SA, van Leeuwen SP, Perfluoroalkyl and polyfluoroalkyl substances in the environment: terminology, classification, and origins, Integr. Environ. Assess. Manag 7 (2011) 513–541. 21793199 10.1002/ieam.258 PMC3214619

[12] Carstens KE, Freudenrich T, Wallace K, Choo S, Carpenter A, Smeltz M, Clifton MS, Henderson WM, Richard AM, Patlewicz G, Evaluation of per-and polyfluoroalkyl substances (PFAS) in vitro toxicity testing for developmental neurotoxicity, Chem. Res. Toxicol 36 (2023) 402–419. 36821828 10.1021/acs.chemrestox.2c00344 PMC10249374

[13] Patlewicz G, Judson RS, Williams AJ, Butler T Jr Barone S Jr., Carstens KE, Cowden J, Dawson JL, Degitz SJ, Fay K, Henry TR, Lowit A, Padilla S, Paul Friedman K, Phillips MB, Turk D, J F W, Wetmore BA, Thomas RS, Development of chemical categories for per-and polyfluoroalkyl substances (PFAS) and the proof-of-concept approach to the identification of potential candidates for tiered toxicological testing and human health assessment, Comput. Toxicol 31 (2024) 100327. 40547594 10.1016/j.comtox.2024.100327 PMC12181936

[14] Wang LX, Yang T, Liu XL, Liu JX, Liu WX, Critical evaluation and meta-analysis of ecotoxicological data on per-and polyfluoroalkyl substances (PFAS) in freshwater species, Environ. Sci. Technol 58 (2024) 17555–17566. 39316471 10.1021/acs.est.4c04818

[15] Zhang W, Liang YN, Interactions between Lemna minor (common duckweed) and PFAS intermediates: perfluorooctanesulfonamide (PFOSA) and 6: 2 fluorotelomer sulfonate (6: 2 FTSA), Chemosphere 276 (2021) 130165. 33714153 10.1016/j.chemosphere.2021.130165

[16] Pietrini F, Passatore L, Fischetti E, Carloni S, Ferrario C, Polesello S, Zacchini M, Evaluation of morpho-physiological traits and contaminant accumulation ability in Lemna minor L. treated with increasing perfluorooctanoic acid (PFOA) concentrations under laboratory conditions, Sci. Total Environ 695 (2019) 133828. 31419689 10.1016/j.scitotenv.2019.133828

[17] Hekster FM, Laane RW, De Voogt P, Environmental and toxicity effects of perfluoroalkylated substances, Rev. Environ. Contam. Toxicol 179 (2003) 99–121. 15366585 10.1007/0-387-21731-2_4

[18] Ma T, Ye C, Wang T, Li X, Luo Y, Toxicity of per-and polyfluoroalkyl substances to aquatic invertebrates, planktons, and microorganisms, Int. J. Environ. Res. Public Health 19 (2022) 16729. 36554610 10.3390/ijerph192416729 PMC9779086

[19] Peritore AF, Gugliandolo E, Cuzzocrea S, Crupi R, Britti D, Current review of increasing animal health threat of per-and polyfluoroalkyl substances (PFAS): harms, limitations, and alternatives to manage their toxicity, Int. J. Mol. Sci 24 (2023) 11707. 37511474 10.3390/ijms241411707 PMC10380748

[20] Drost W, Matzke M, Backhaus T, Heavy metal toxicity to Lemna minor: studies on the time dependence of growth inhibition and the recovery after exposure, Chemosphere 67 (2007) 36–43. 17157350 10.1016/j.chemosphere.2006.10.018

[21] Wang MC, Rivenbark K, Gong J, Wright FA, Phillips TD, Application of edible montmorillonite clays for the adsorption and detoxification of microcystin, ACS Appl. Bio Mater 4 (2021) 7254–7265. 10.1021/acsabm.1c00779 PMC8570584 34746680

[22] U.S. Environmental Protection Agency. OCSPP 859.4400: Background Concentration of Microorganisms in Soil; Office of Chemical Safety and Pollution Prevention.

[23] Verma S, Mezgebe B, Hejase CA, Sahle-Demessie E, Nadagouda MN, Photodegradation and photocatalysis of per-and polyfluoroalkyl substances (PFAS): a review of recent progress, mater 2 (2024) 1–12, 100077. 10.1016/j.nxmate.2023.100077 PMC11151751 38840836

[24] Turchi CS, Ollis DF, Photocatalytic degradation of organic water contaminants: mechanisms involving hydroxyl radical attack, J. Catal 122 (1990) 178–192.

[25] Chen Y, Bhati M, Walls BW, Wang B, Wong MS, Senftle TP, Mechanistic insight into the photo-oxidation of perfluorocarboxylic acid over boron nitride, Environ. Sci. Technol 56 (2022) 8942–8952. 35617117 10.1021/acs.est.2c01637

[26] Lashuk B, Pineda M, AbuBakr S, Boffito D, Yargeau V, Application of photocatalytic ozonation with a WO_3_/TiO_2_ catalyst for PFAS removal under UVA/visible light, Sci. Total Environ 843 (2022) 157006. 35779716 10.1016/j.scitotenv.2022.157006

[27] Schulz K, Silva MR, Klaper R, Distribution and effects of branched versus linear isomers of PFOA, PFOS, and PFHxS: a review of recent literature, Sci. Total Environ 733 (2020) 139186, 139186. 10.1016/j.scitotenv.2020.139186 32474294

[28] Santiago-Cruz HA, Lou ZM, Xu J, Sullivan RC, Bowers BB, Molé RA, Zhang W, Li JH, Yuan JS, Dai SY, Carbon Adsorbent Properties Impact Hydrated Electron Activity and Perfluorocarboxylic Acid (PFCA) Destruction, ACS ES&T Eng 4, 2220–2233. 10.1021/acsestengg.4c00211 PMC11406532 39296420

[29] Sansotera M, Persico F, Rizzi V, Panzeri W, Pirola C, Bianchi CL, Mele A, Navarrini W, The effect of oxygen in the photocatalytic oxidation pathways of perfluorooctanoic acid, J. Fluor Chem 179 (2015) 159–168.

[30] Gomez-Ruiz B, Ribao P, Diban N, Rivero MJ, Ortiz I, Urtiaga A, Photocatalytic degradation and mineralization of perfluorooctanoic acid (PFOA) using a composite TiO_2_-rGO catalyst, J. Hazard Mater 344 (2018) 950–957. 29197229 10.1016/j.jhazmat.2017.11.048

