# Supplementary material for: Photocatalytic degradation of perfluorooctanoic acid under ambient conditions validated by duckweed as a sensitive ecotoxicity assay
Source: J Hazard Mater Org. Author manuscript; Available in PMC 2026 Jul 24. (PMC13392970; doi:10.1016/j.hazmo.2026.100016)
Supplement: Supplementary material 1 [file NIHMS2191960-supplement-Supplementary_material_1.docx]

**Supplementary Materials**

**Photocatalytic Degradation of Perfluorooctanoic Acid Under Ambient Conditions Validated by Duckweed as a Sensitive Ecotoxicity Assay**

Wan Zhang ^a+^, Meichen Wang ^b+^, Vincent Xu ^c^, Johnson O. Oladele ^d^, Yohannes H. Rezenom ^e^, Timothy D. Phillips ^d*^, Susie Y. Dai ^a,f*^

^a^ Department of Plant Pathology and Microbiology, Texas A&M University, College Station, TX 77843

^b^ Department of Environmental Health Sciences, University of Massachusetts Amherst, Amherst, Massachusetts 01003, USA

^c^ Department of Energy, Chemical and Environmental Engineering, Washington University in St Louis, MO, 63130

^d^ Department of Veterinary Physiology and Pharmacology, College of Veterinary Medicine & Biomedical Sciences, Texas A&M University, College Station, Texas 77845, USA

^e^ Department of Chemistry, Texas A&M University, College Station, TX 77843

^f^ Department of Chemical and Biomedical Engineering, University of Missouri, Columbia, MO 65211

^+^ The two authors contributed equally to the work

^*^Corresponding authors:

Dr. Timothy Phillips: tphillips@cvm.tamu.edu

Dr. Susie Y. Dai: sydai@missouri.edu

Table of Contents

1. **The detection limitation for the high-resolution mass spectrometry 2**
2. **EC_50_ of PFOA, PFHpA, and PFHxA 3**
3. **Degradation product's peak areas 4**
4. **The LC-triple quadrupole mass spectrometry analysis of 7.5 ppm PFOA 4**
5. **Analysis of PFOA in the absence of photocatalyst under solar light treatment 5**
6. **Confidence interval for different water matrices 5**
7. **Fitted parameters for different water matrices 6**
8. **Individual subfigures of Fig.3 7**
9. Individual subfigures of Fig.5 **9**

Table S1. The detection limitation for the high-resolution mass spectrometry

| **PFDA Conc.** | **Peak Area** | | | **Curve** |
| --- | --- | --- | --- | --- |
| **ng/L** |  |  |  |  |
| 0.515 | 8.83E+06 | 8.69E+06 | 8.53E+06 | 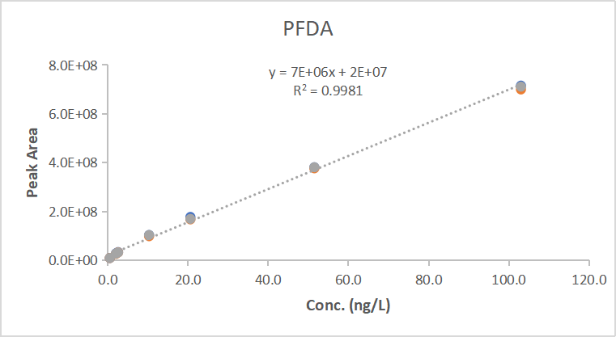 |
| 2.06 | 2.81E+07 | 2.57E+07 | 2.69E+07 |  |
| 2.575 | 3.30E+07 | 3.18E+07 | 3.15E+07 |  |
| 10.3 | 1.03E+08 | 9.80E+07 | 1.03E+08 |  |
| 20.6 | 1.77E+08 | 1.67E+08 | 1.69E+08 |  |
| 51.5 | 3.81E+08 | 3.77E+08 | 3.81E+08 |  |
| 103 | 7.16E+08 | 7.00E+08 | 7.11E+08 |  |
| **PFNA Conc.** | **Peak Area** | | | **Curve** |
| **ng/L** |  |  |  |  |
| 0.24 | 3.45E+06 | 3.31E+06 | 3.16E+06 | 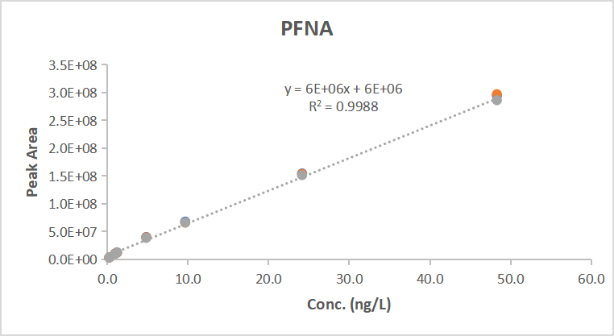 |
| 0.97 | 1.05E+07 | 1.04E+07 | 9.74E+06 |  |
| 1.21 | 1.25E+07 | 1.20E+07 | 1.22E+07 |  |
| 4.83 | 4.02E+07 | 3.96E+07 | 3.87E+07 |  |
| 9.66 | 6.79E+07 | 6.61E+07 | 6.64E+07 |  |
| 24.15 | 1.55E+08 | 1.54E+08 | 1.52E+08 |  |
| 48.30 | 2.95E+08 | 2.97E+08 | 2.86E+08 |  |
| **PFOA Conc.** | **Peak Area** | | | **Curve** |
| **ng/L** |  |  |  |  |
| 0.88 | 1.30E+07 | 1.21E+07 | 1.19E+07 | 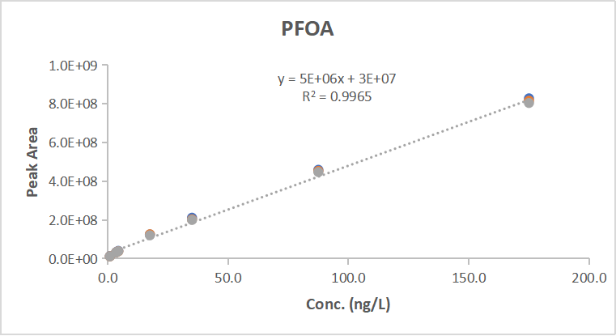 |
| 3.5 | 3.37E+07 | 3.28E+07 | 3.25E+07 |  |
| 4.38 | 4.03E+07 | 3.82E+07 | 3.85E+07 |  |
| 17.50 | 1.25E+08 | 1.26E+08 | 1.21E+08 |  |
| 35.00 | 2.11E+08 | 2.03E+08 | 2.00E+08 |  |
| 87.50 | 4.59E+08 | 4.53E+08 | 4.48E+08 |  |
| 175.00 | 8.28E+08 | 8.16E+08 | 8.05E+08 |  |
| **PFHpA Conc.** | **Peak Area** | | | **Curve** |
| **ng/L** |  |  |  |  |
| 0.59 | 5.52E+06 | 5.33E+06 | 5.19E+06 | 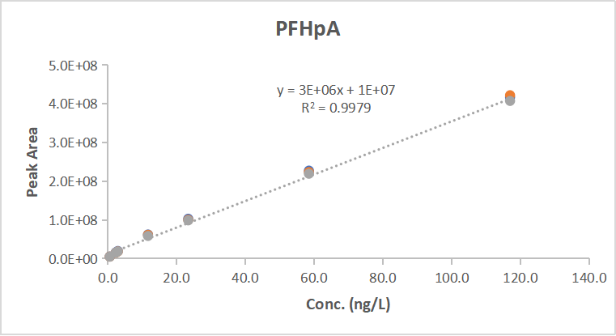 |
| 2.34 | 1.63E+07 | 1.54E+07 | 1.55E+07 |  |
| 2.93 | 1.99E+07 | 1.87E+07 | 1.88E+07 |  |
| 11.70 | 6.24E+07 | 6.16E+07 | 5.87E+07 |  |
| 23.40 | 1.03E+08 | 1.00E+08 | 9.90E+07 |  |
| 58.50 | 2.28E+08 | 2.24E+08 | 2.19E+08 |  |
| 117.00 | 4.17E+08 | 4.22E+08 | 4.08E+08 |  |
| **PFHxA Conc.** | **Peak Area** | | | **Curve** |
| **ng/L** |  |  |  |  |
| 0.57 | 4.24E+06 | 3.66E+06 | 4.12E+06 | 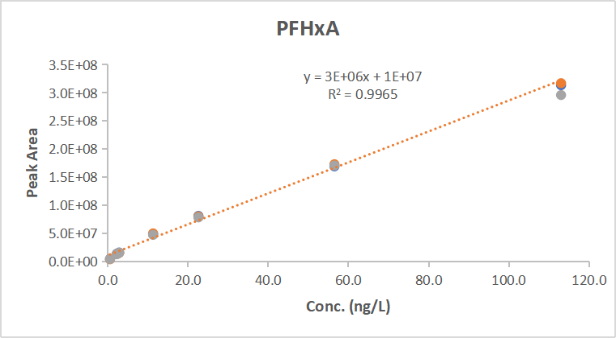 |
| 2.26 | 1.35E+07 | 1.29E+07 | 1.26E+07 |  |
| 2.83 | 1.56E+07 | 1.49E+07 | 1.51E+07 |  |
| 11.30 | 4.85E+07 | 4.97E+07 | 4.71E+07 |  |
| 22.60 | 8.08E+07 | 7.98E+07 | 7.82E+07 |  |
| 56.50 | 1.69E+08 | 1.73E+08 | 1.71E+08 |  |
| 113.00 | 3.14E+08 | 3.17E+08 | 2.96E+08 |  |
| **PFPeA Conc.** | **Peak Area** | | | **Curve** |
| **ng/L** |  |  |  |  |
| 1.88 | 4.52E+06 | 4.16E+06 | 6.98E+06 | 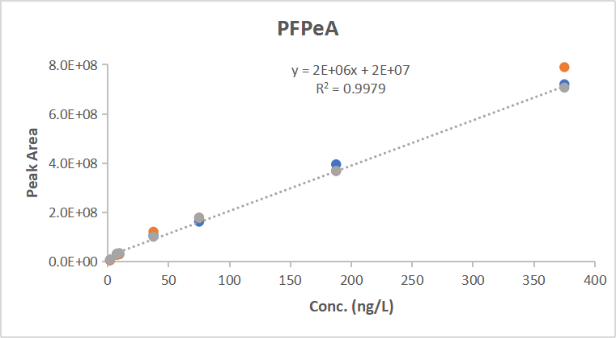 |
| 7.5 | 3.06E+07 | 2.62E+07 | 2.99E+07 |  |
| 9.38 | 2.89E+07 | 2.86E+07 | 3.36E+07 |  |
| 37.50 | 1.05E+08 | 1.20E+08 | 9.99E+07 |  |
| 75.00 | 1.61E+08 | 1.78E+08 | 1.77E+08 |  |
| 187.50 | 3.95E+08 | 3.67E+08 | 3.68E+08 |  |
| 375.00 | 7.21E+08 | 7.90E+08 | 7.07E+08 |  |
| **PFBA Conc.** | **Peak Area** | | | **Curve** |
| **ng/L** |  |  |  |  |
| 3.84 | 4.57E+05 | 6.28E+05 | 7.24E+05 | 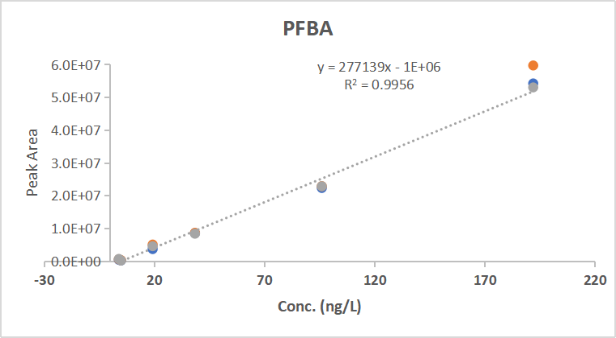 |
| 4.80 | 2.32E+05 | 3.58E+05 | 2.41E+05 |  |
| 19.20 | 3.73E+06 | 5.11E+06 | 4.61E+06 |  |
| 38.40 | 8.59E+06 | 8.70E+06 | 8.43E+06 |  |
| 96.00 | 2.24E+07 | 2.29E+07 | 2.29E+07 |  |
| 192.00 | 5.43E+07 | 5.98E+07 | 5.31E+07 |  |

Table S2. EC_50_ of PFOA, PFHpA, and PFHxA

| EC50 (ppm) | PFOA | PFHpA | PFHxA |
| --- | --- | --- | --- |
| Frond number | 6.51±2.01 | 40.6±5.15 | 25.1±4.8 |
| Surface area | 7.14±0.71 | 60.2±10.2 | 131±12.6 |
| Chlorophyll | 8.23±1.35 | 81.5±21.7 | 482±22.5 |

Table S3. Degradation product's peak areas identified by high-resolution mass spectrometry

| Sample | 7.5 ppm C_7_F_15_COOH (area) | | | | | 40 ppm C_6_F_13_COOH (area) | | | | |
| --- | --- | --- | --- | --- | --- | --- | --- | --- | --- | --- |
|  | 0 h | 0.5 h | 1 h | 3 h | 6 h | 0 h | 0.5 h | 1 h | 3 h | 6 h |
| C_7_F_15_COOH (PFOA) | 6.91×10^9^ | 6.29×10^9^ | 6.09×10^9^ | 5.59×10^9^ | 5.47×10^9^ | - | - | - | - | - |
| C_6_F_13_COOH  (PFHpA) | - | 3.6×10^7^ | 5.5×10^7^ | 5.3×10^8^ | 6.6×10^8^ | 1.1×10^10^ | 1.0×10^10^ | 1.0×10^10^ | 1.0×10^10^ | 9.76×10^9^ |
| C_5_F_11_COOH (PFHxA) | - | 8.66×10^6^ | 7.34×10^6^ | 4.23×10^7^ | 6.12×10^7^ | - | 3.64×10^7^ | 2.32×10^8^ | 4.27×10^8^ | 1.15×10^9^ |
| C_4_F_9_COOH (PFPeA) | - | - | - | 6.76×10^6^ | 1.04×10^7^ | - | 6.23×10^5^ | 7.53×10^6^ | 8.94×10^7^ | 8.27×10^7^ |
| C_3_F_7_COOH  (PFBA) | - | - | - | - | 4.32×10^5^ | - | 5.05×10^5^ | 1.67×10^6^ | 8.82×10^6^ | 1.09×10^7^ |

Table S4. The LC-triple quadrupole mass spectrometry analysis of degradation products from solar light-treated 7.5 ppm PFOA (C_7_F_15_COOH) in LC water for 6 h

| Time (hour) | PFOA (ppm) | PFHpA (ppm) | PFHxA (ppm) |
| --- | --- | --- | --- |
| 0 | 7.6 ± 0.2 | 0 | 0 |
| 0.5 | 6.9 ± 0.1 | 0 | 0 |
| 1 | 6.9 ± 0.2 | 0 | 0 |
| 3 | 6.1 ± 0.2 | 0.4 ± 0.0 | 0.19 ± 0.0 |
| 6 | 5.5 ± 0.2 | 0.5 ± 0.0 | 0.19 ± 0.0 |

Table S5. The LC-triple quadrupole mass spectrometry analysis of LC water containing 7.5 ppm PFOA in the absence of photocatalyst under solar light treatment

| Time (hour) | PFOA (ppm) | PFHpA (ppm) | PFHxA (ppm) |
| --- | --- | --- | --- |
| 0 | 7.3 ± 0.1 | 0 | 0 |
| 0.5 | 7.7 ± 0.3 | 0 | 0 |
| 1 | 7.5 ± 0.0 | 0 | 0 |
| 3 | 7.7 ± 0.0 | 0 | 0 |
| 6 | 7.5 ± 0.2 | 0 | 0 |

Table S6 Confidence interval for different water matrices

| Parameter | Lower Bound | Predicted Value | Upper Bound |
| --- | --- | --- | --- |
| LC-Water | | | |
| 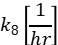 | 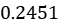 | 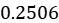 | 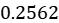 |
| 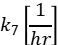 | 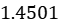 | 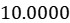 | 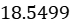 |
| 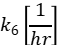 | 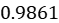 | 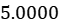 | 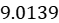 |
| Tap Water | | | |
| 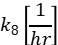 | 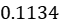 | 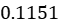 | 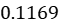 |
| 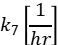 | 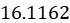 | 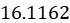 | 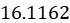 |
| 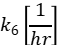 | 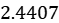 | 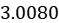 | 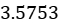 |
| Rainwater | | | |
| 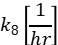 | 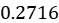 | 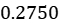 | 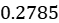 |
| 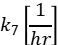 | 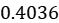 | 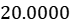 | 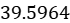 |
| 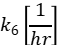 | 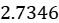 | 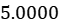 | 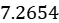 |
| Groundwater | | | |
| 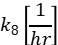 | 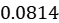 | 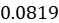 | 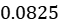 |
| 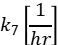 | 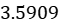 | 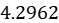 | 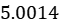 |
| 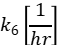 | 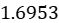 | 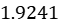 | 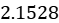 |
| Wastewater | | | |
| 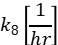 | 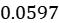 | 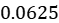 | 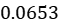 |
| 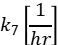 | 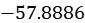 | 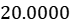 | 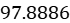 |
| 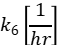 | 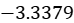 | 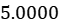 | 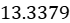 |

Table S7 Fitted parameters for different water matrices

| PFOA R^2^ | PFHpA R^2^ | PFHxA R^2^ | F^-^ R^2^ |
| --- | --- | --- | --- |
| LC-Water | | | |
| 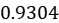 | 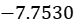 | 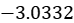 | 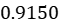 |
| Tap Water | | | |
| 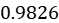 | 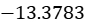 | 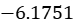 | 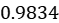 |
| Rainwater | | | |
| 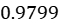 | 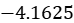 | 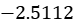 | 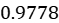 |
| Groundwater | | | |
| 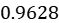 | 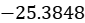 | 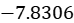 | 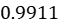 |
| Wastewater | | | |
| 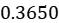 | 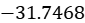 | 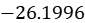 | 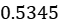 |


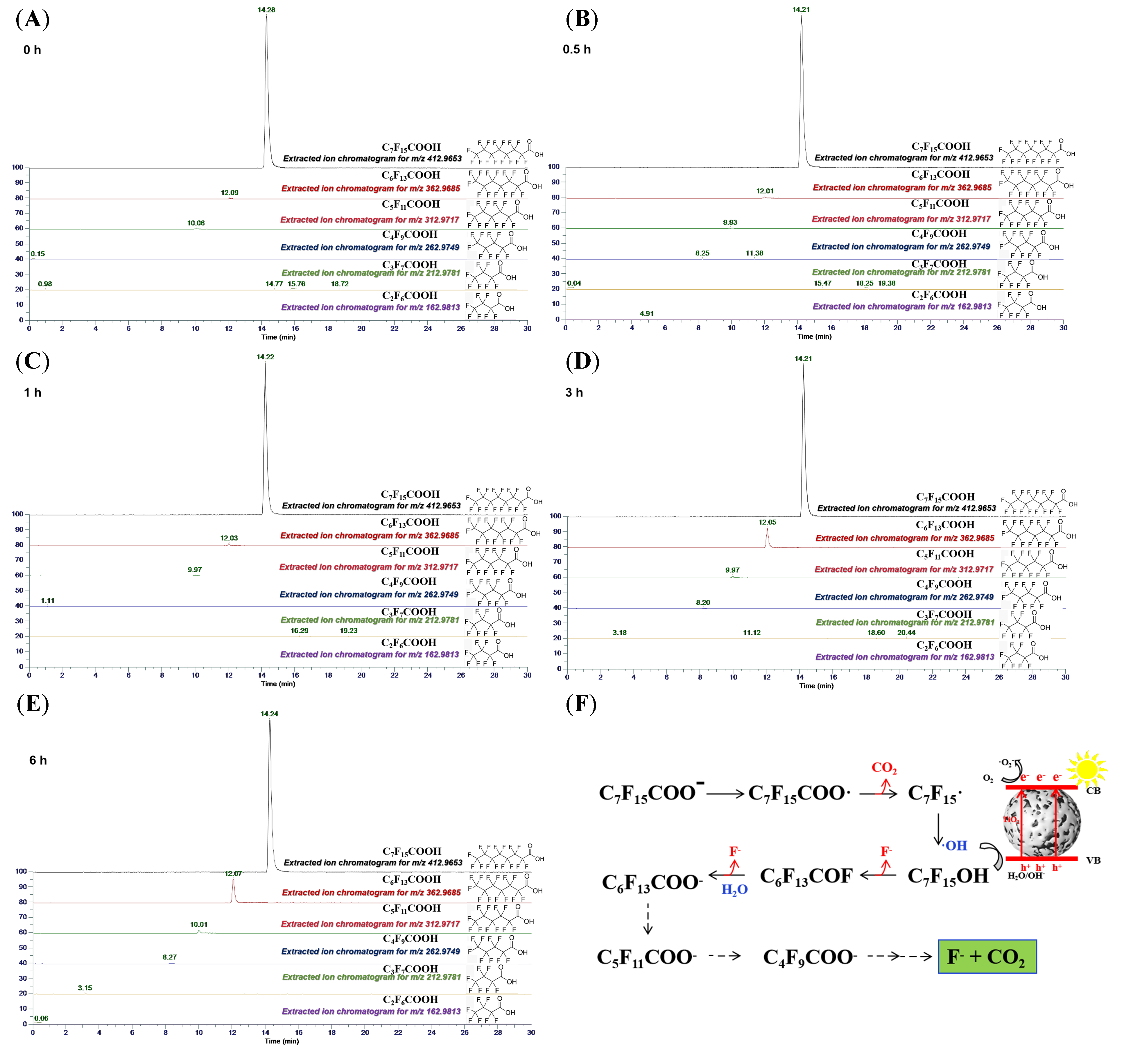

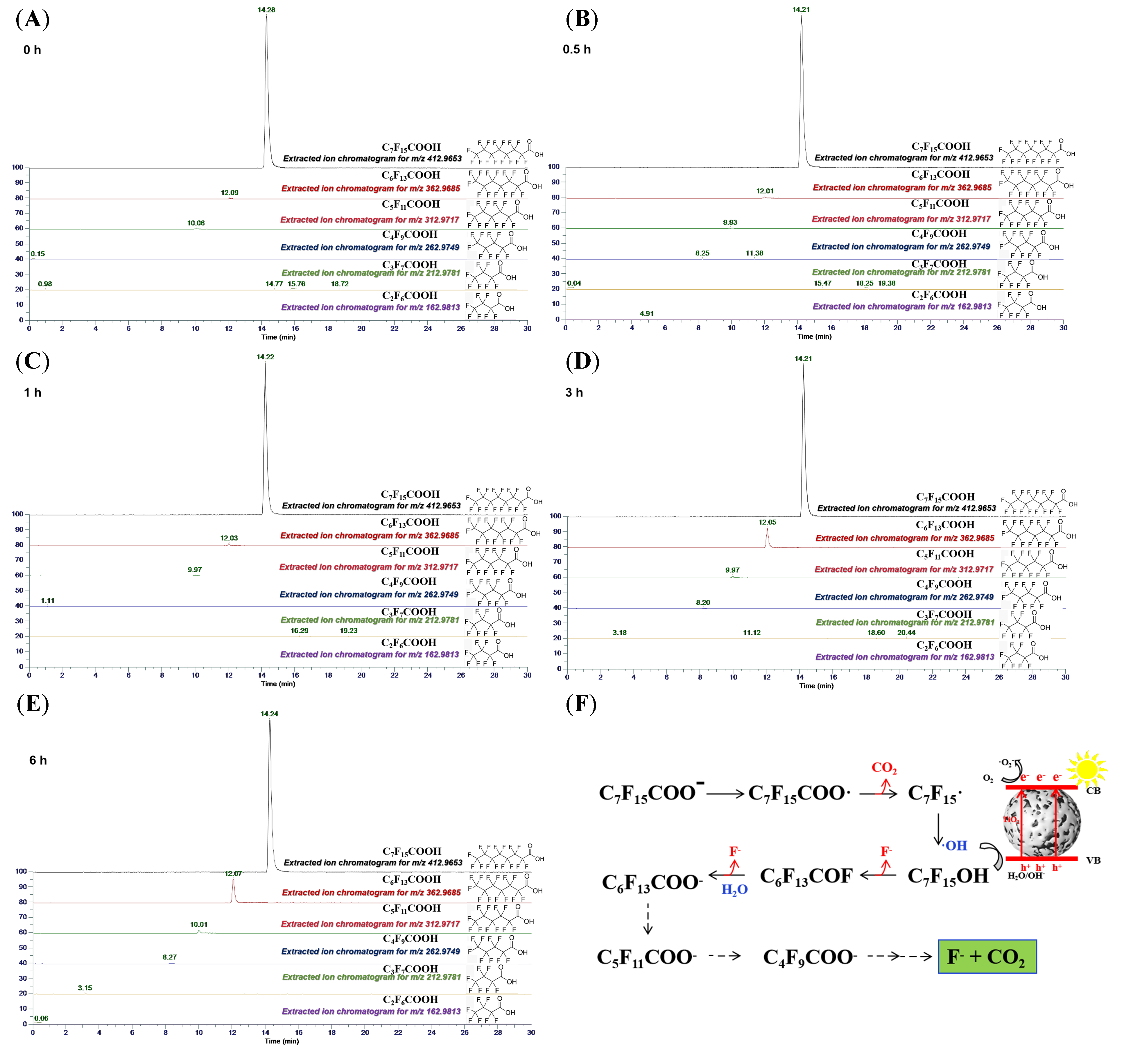

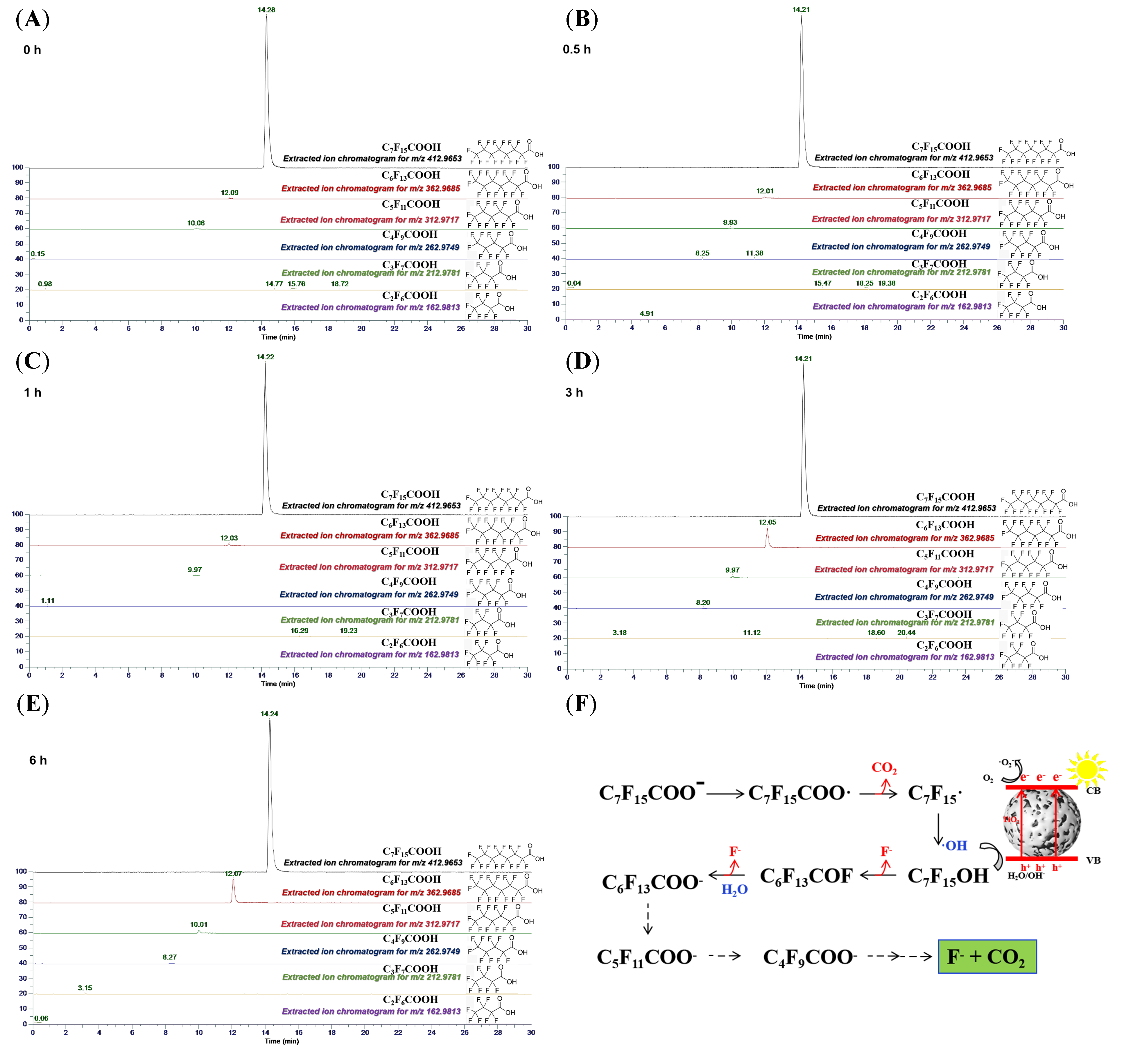

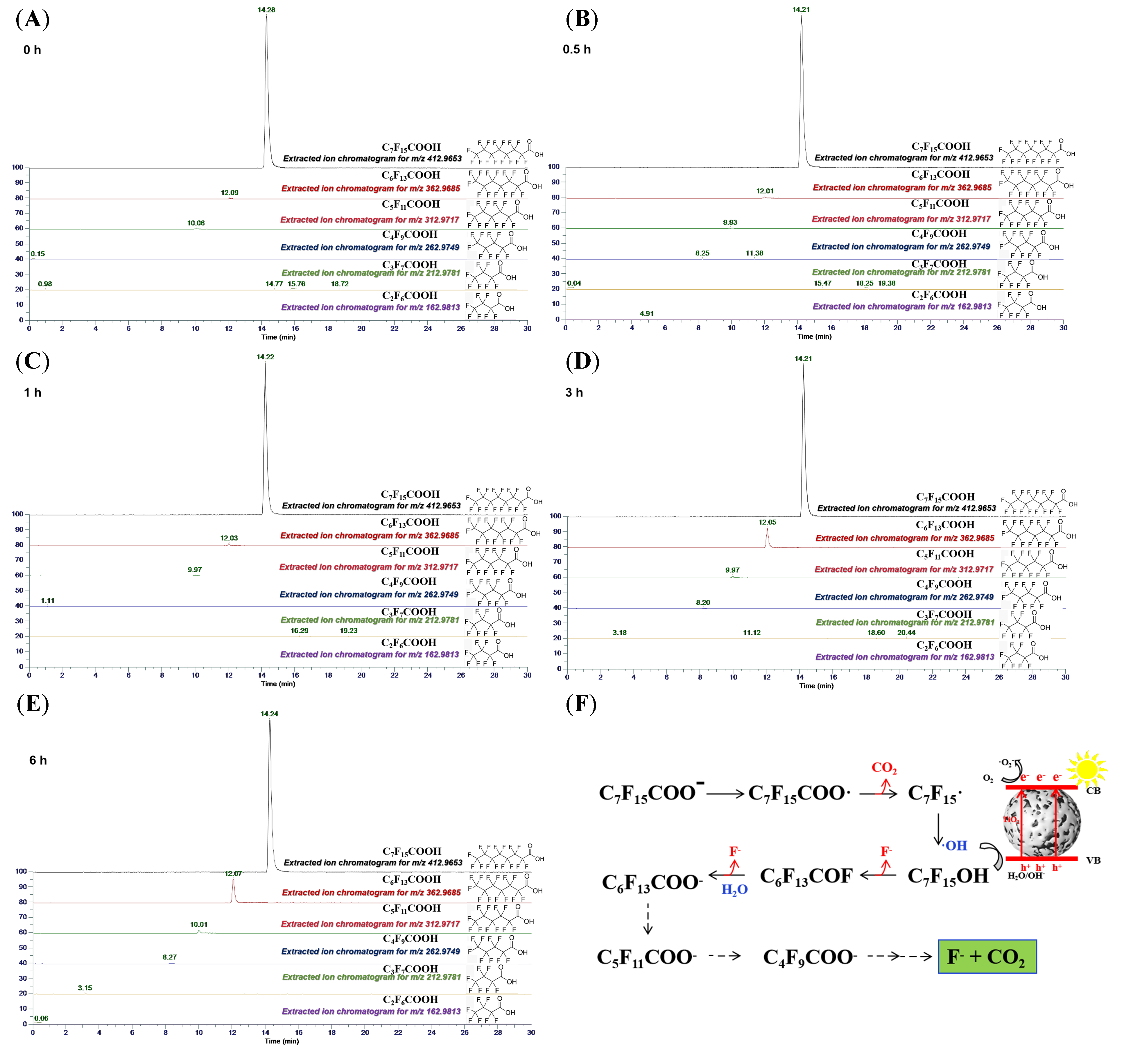

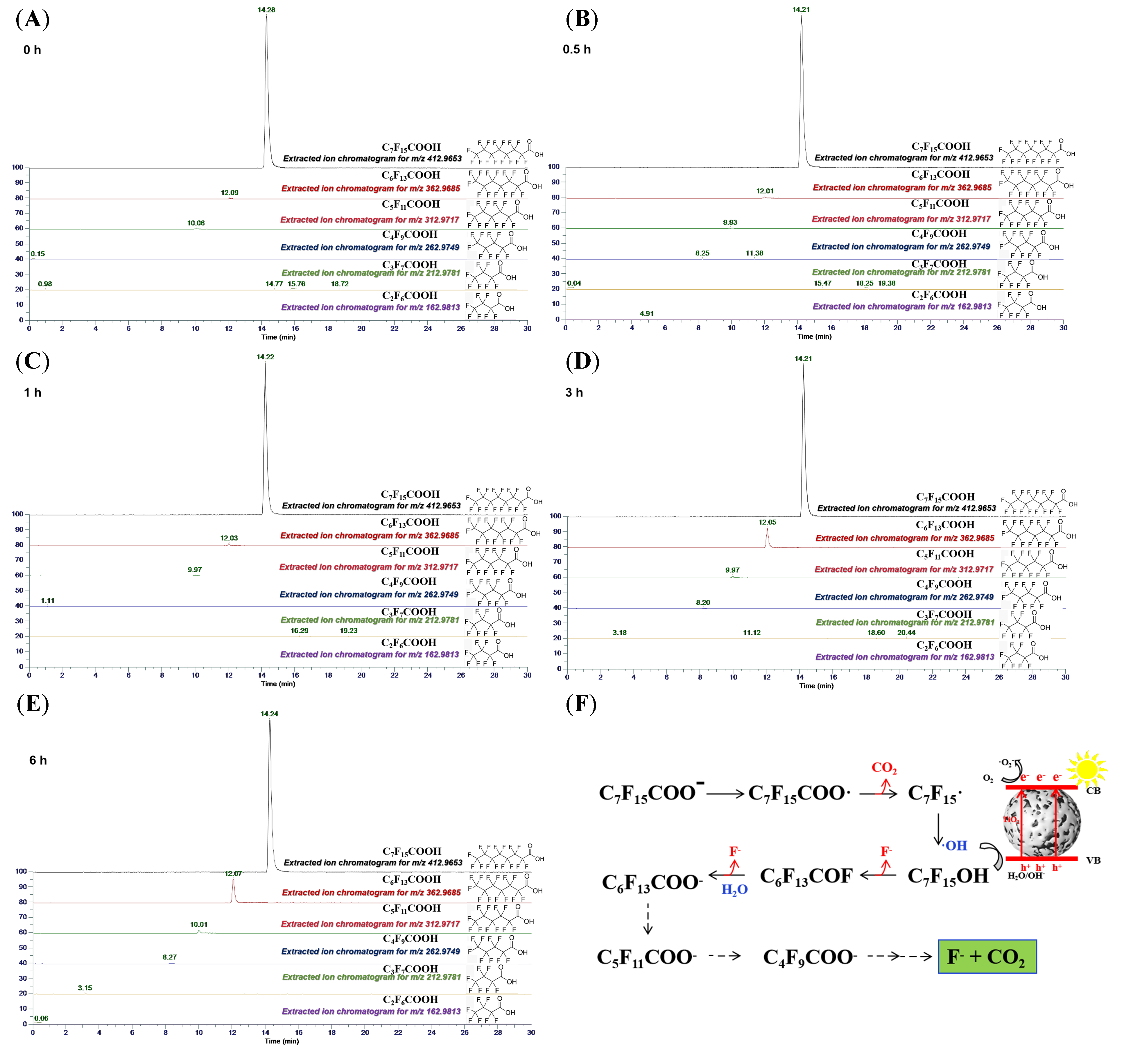
 Fig. S1. Individual subfigures of Fig. 3


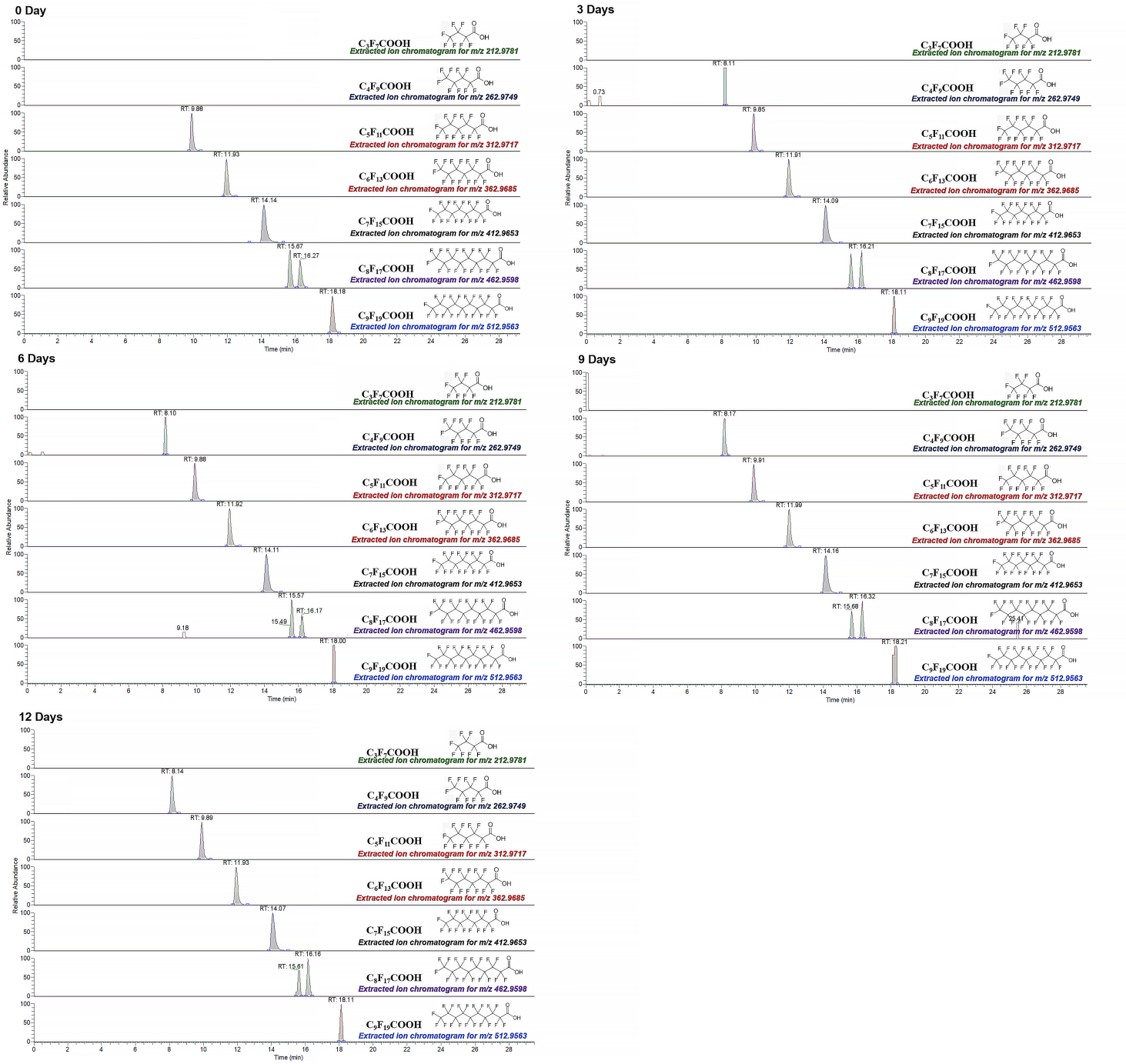


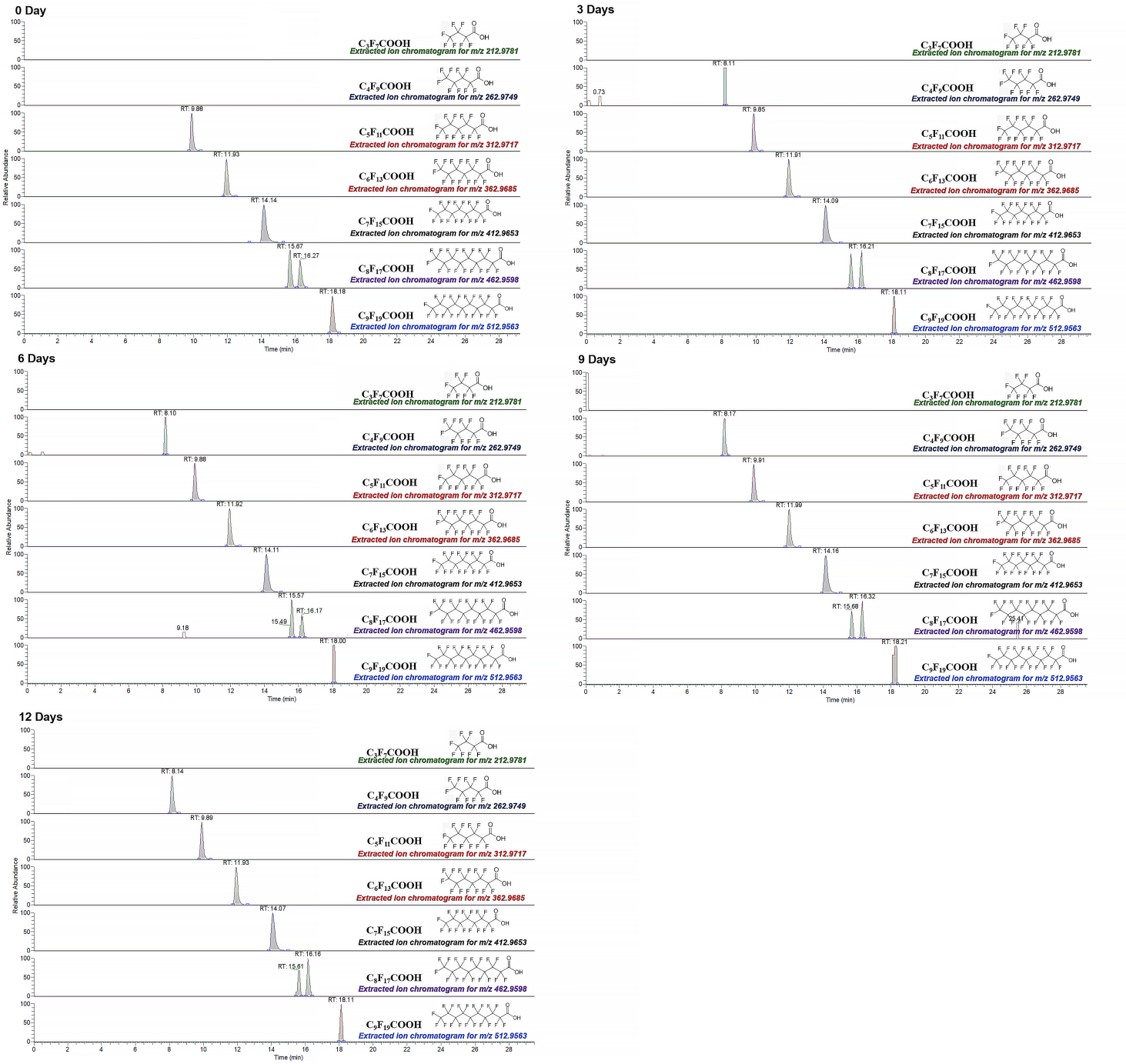


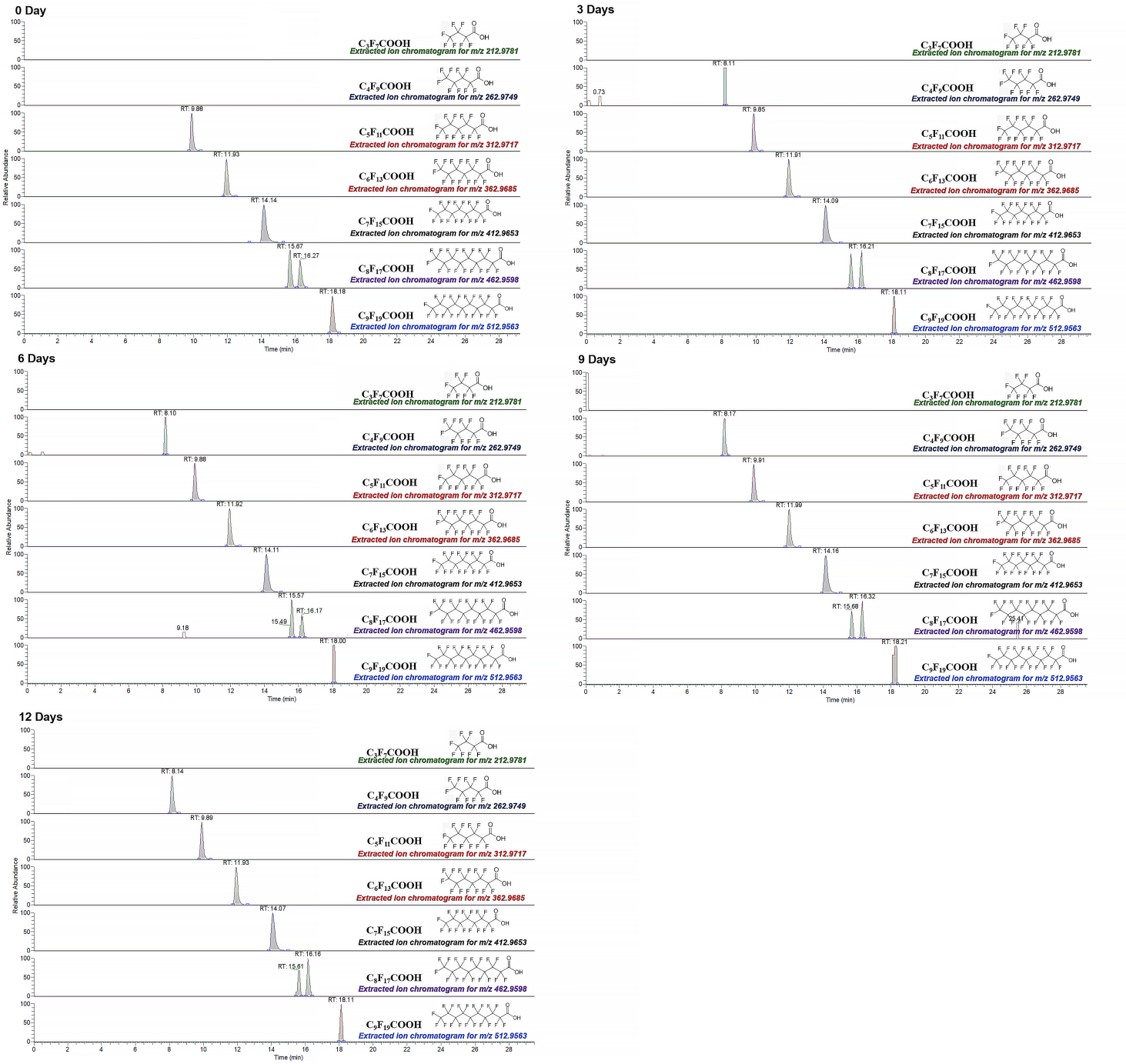


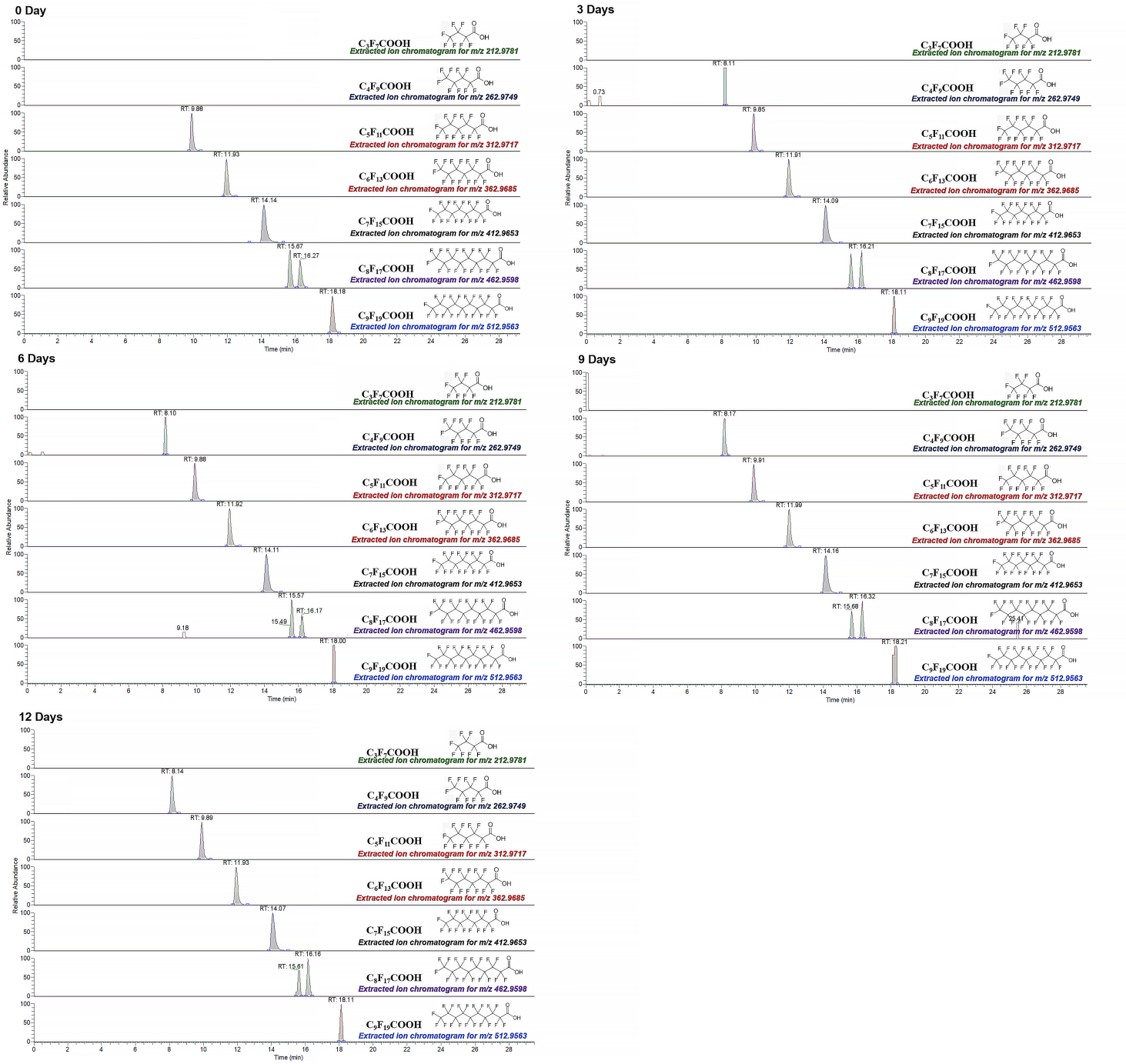


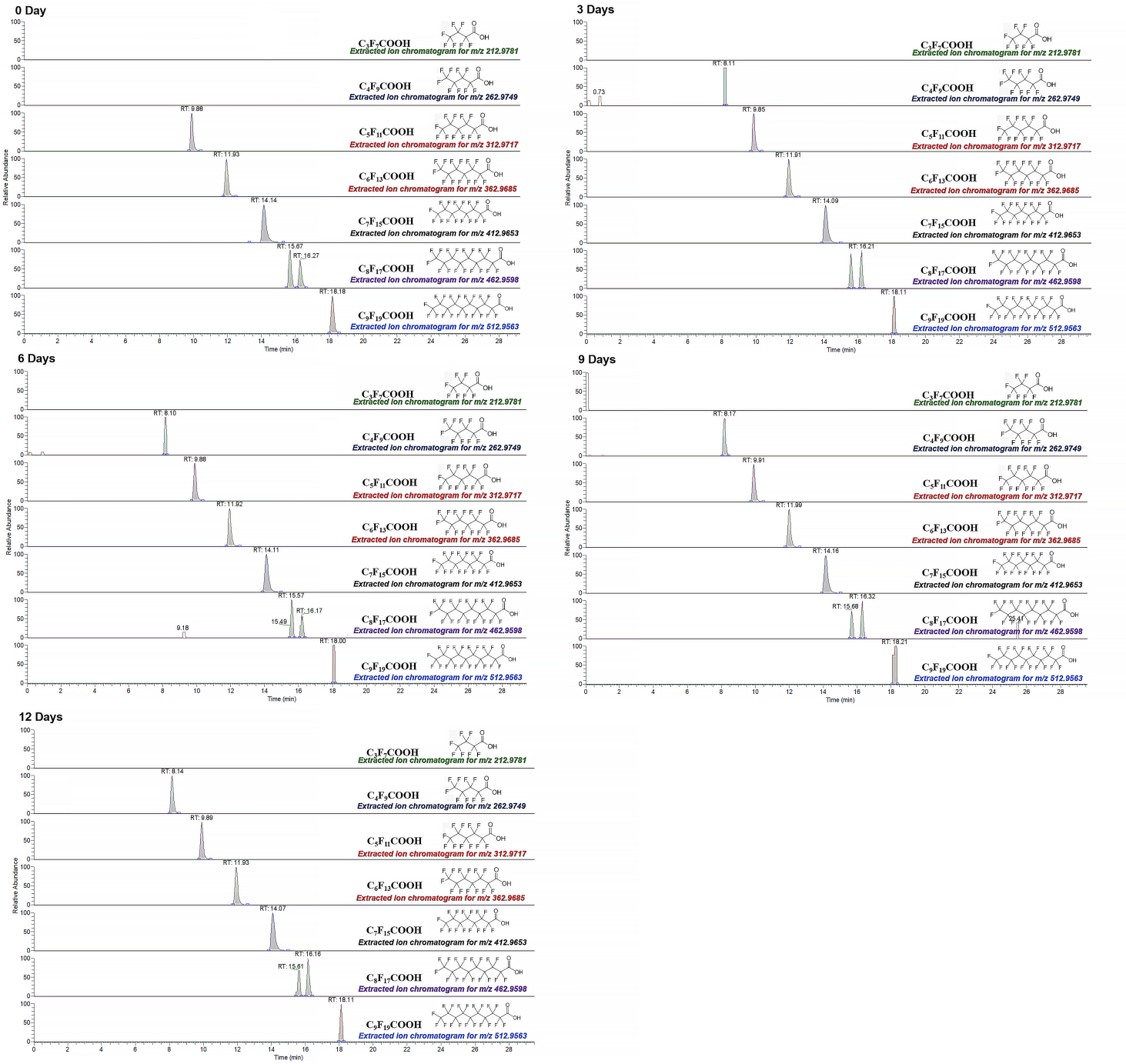


Fig. S2. Individual subfigures of Fig. 5
